# Structural basis for HIV-1 antagonism of host APOBEC3G via Cullin E3 ligase

**DOI:** 10.1126/sciadv.ade3168

**Published:** 2023-01-04

**Authors:** Fumiaki Ito, Ana L. Alvarez-Cabrera, Shiheng Liu, Hanjing Yang, Anna Shiriaeva, Z. Hong Zhou, Xiaojiang S. Chen

**Affiliations:** ^1^ Molecular and Computational Biology, Departments of Biological Sciences, University of Southern California, Los Angeles, CA, USA.; ^2^ Department of Microbiology, Immunology and Molecular Genetics, University of California, Los Angeles (UCLA), Los Angeles, CA, USA.; ^3^ California NanoSystems Institute, UCLA, Los Angeles, CA, USA.; ^4^ Department of Biological Chemistry, UCLA, Los Angeles, CA, USA.; ^5^ Genetic, Molecular, and Cellular Biology Program, Keck School of Medicine, University of Southern California, Los Angeles, CA, USA.; ^6^ Norris Comprehensive Cancer Center, University of Southern California, Los Angeles, CA, USA.; ^7^ Center of Excellence in NanoBiophysics, University of Southern California, Los Angeles, CA, USA.

## Abstract

Human APOBEC3G (A3G) is a virus restriction factor that inhibits HIV-1 replication and triggers lethal hypermutation on viral reverse transcripts. HIV-1 viral infectivity factor (Vif) breaches this host A3G immunity by hijacking a cellular E3 ubiquitin ligase complex to target A3G for ubiquitination and degradation. The molecular mechanism of A3G targeting by Vif-E3 ligase is unknown, limiting the antiviral efforts targeting this host-pathogen interaction crucial for HIV-1 infection. Here, we report the cryo–electron microscopy structures of A3G bound to HIV-1 Vif in complex with T cell transcription cofactor CBF-β and multiple components of the Cullin-5 RING E3 ubiquitin ligase. The structures reveal unexpected RNA-mediated interactions of Vif with A3G primarily through A3G’s noncatalytic domain, while A3G’s catalytic domain is poised for ubiquitin transfer. These structures elucidate the molecular mechanism by which HIV-1 Vif hijacks the host ubiquitin ligase to specifically target A3G to establish infection and offer structural information for the rational development of antiretroviral therapeutics.

## Teaser

Structure of HIV-1 Vif and E3 ligase in complex with host APOBEC3G illuminates a basis for immune evasion by HIV.

## INTRODUCTION

The arms race between lentiviruses and their hosts manifests in our fight against HIV/AIDS as HIV-1 viral infectivity factor (Vif) and host APOBEC3G (A3G) evolve to take control of each other. Human A3G (hA3G) potently restricts Vif-deficient HIV-1 (HIV-1ΔVif) replication mainly by hypermutating single-stranded DNA (ssDNA) reverse transcripts of the viral genome (*1*–*4*). A3G contains two cytidine deaminase (CD) domains with complementary roles in HIV restriction, the N-terminal noncatalytic CD1, and the C-terminal catalytic CD2 domains. The CD1 has a high affinity for RNA, which is a prerequisite for A3G to be packaged into budding HIV virions (*5*, *6*). The CD2 catalytically converts cytosine base into uracil in ssDNA and is responsible for triggering G-to-A hypermutation in the complementary DNA (cDNA) reverse transcripts of HIV genome (*7*–*11*). A3G also has deaminase-independent HIV restriction activity through inhibiting viral replication possibly by an RNA binding–related roadblock mechanism or direct inhibition of HIV reverse transcriptase (*8*, *9*, *12*, *13*). In addition, newly synthesized A3G can associate with RNA and subsequently form high–molecular mass (HMM) ribonucleoprotein complexes in cells over time (*14*–*16*). HIV-1 encodes Vif protein that can effectively antagonize A3G by inducing polyubiquitination of A3G for subsequent proteasomal degradation (*17*–*19*). In this process, Vif hijacks host Cullin-5 (CUL5) RING E3 ubiquitin ligase complex containing Elongin B/Elonging C (ELOB/ELOC) adaptor and zinc finger RING-box protein 2 (RBX2) (*20*). Vif additionally recruits a T cell transcription cofactor, the core binding factor beta (CBF-β), into the complex to modulate host transcription (*21*, *22*).

Despite tremendous advancement in the structural understanding of A3G and Vif-E3 ligase subcomplexes (*23*–*26*), the molecular mechanisms by which Vif-E3 ligase recognizes A3G for destruction remains unclear because of the lack of structures of A3G in complex with Vif and E3 ligase. We report here the atomic structure of a primate A3G bound to HIV-1 Vif-E3 ligase complex. The structure reveals the molecular details of A3G recognition by Vif-E3 ligase and how A3G in the complex is oriented for ubiquitination and subsequent degradation.

## RESULTS

### Reconstitution of functional A3G-Vif complex in vitro

**Fig. 1.**
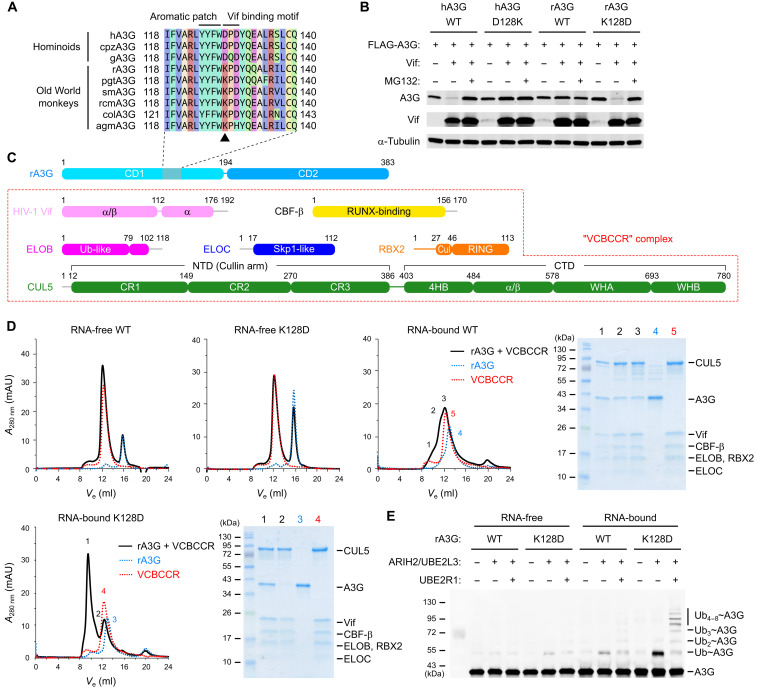
Reconstitution of the functional A3G-Vif complex. (**A**) Alignment of primate A3G sequences. At amino acid position 128 (marked by a triangle) within the Vif binding motif, hominoids have aspartate (D128), whereas Old World monkeys have lysine (K128). cpzA3G, chimpanzee; gA3G, gorilla; pgtA3G, pig-tailed macaque; smA3G, sooty mangabey; rcmA3G, red-capped macaque; colA3G, Eastern black-and-white colobus monkey; agmA3G, African green monkey. (**B**) Vif-mediated degradation assay of A3G. Western blots show that the steady-state level of hA3G in human embryonic kidney 293T cells was lowered in the presence of HIV-1 Vif but restored by a proteasome inhibitor *N*-carbobenzyloxy-l-leucyl-l-leucyl-l-leucinal (MG132). D128K mutation restored the hA3G level in the presence of HIV-1 Vif. The rA3G level was unchanged in the presence of HIV-1 Vif, but D128K mutation reduced the rA3G level. (**C**) Design of the expression constructs for the seven-protein complex of A3G-VCBCCR. CR, Cullin repeat; 4HB, four-helix bundle; WH, winged helix; thin gray lines, not included in the construct. (**D**) Binding analysis of A3G and VCBCCR complex by SEC. A major peak shift was observed only for the RNA-bound K128D of rA3G. SDS–polyacrylamide gel electrophoresis gels show the protein components in the indicated SEC fractions. *A*_280_, absorbance at 280 nm; mAU, milli-absorbance unit. (**E**) In vitro rA3G ubiquitination assays. Ubiquitin chain initiation and elongation on the purified RNA-free or RNA-bound rA3G were tested with or without ubiquitination enzymes ARIH2/UBE2L3 and UBE2R1, respectively. Monoubiquitination and polyubiquitination products were the most pronounced in the RNA-bound K128D mutant of rA3G.

To reconstitute the A3G-Vif protein complex in vitro, we explored constructs that allow stable complex formation for structural studies. While full-length hA3G and other primate A3Gs are prone to aggregation, we have previously shown that an engineered A3G from rhesus macaque (rA3G) has improved solubility and stability (*23*, *27*). A3Gs from Old World monkeys (cercopithecoids) are degraded by Vif from their cognate simian immunodeficiency viruses (SIV) but are resistant to HIV-1 Vif (*28*, *29*). This phenotype has been attributed to a single amino acid at position 128, at which A3Gs from hominoids have aspartate (D128) and A3Gs from Old World monkeys have lysine (K128) (Fig. 1A) (*30*–*33*). The polymorphism is located within the -^128^DPD^130^- Vif binding motif (Fig. 1A). Our assay confirmed that K128D single substitution in rA3G rendered the protein highly sensitive to HIV-1 Vif–mediated degradation in a proteasome-dependent manner, while D128K in hA3G rendered the protein highly resistant (Fig. 1B). We next purified the rA3G soluble variant and Vif/CBF-β/ELOB/ELOC/CUL5/RBX2 (VCBCCR) complex for an in vitro binding test (Fig. 1C). rA3G was purified as RNA-bound and RNA-free forms, both of which were tested for binding to the VCBCCR complex by size exclusion chromatography (SEC). The RNA-free form of rA3G wild-type (WT) K128 or K128D mutant showed no obvious peak shift when combined with the VCBCCR complex, indicating no molecular interaction. On the other hand, the RNA-bound form of rA3G K128D showed a substantial peak shift, indicating stable binding to the VCBCCR complex. The RNA-bound form of WT rA3G showed only a minor peak shift, perhaps indicative of weak interaction with the VCBCCR complex. These binding test results suggest that the complex formation between rA3G K128D and VCBCCR is unlikely to be a result of a mere individual RNA binding event (Fig. 1D). An in vitro ubiquitination assay was then performed with RNA-bound and RNA-free forms of rA3G WT K128 and K128D in the presence of Vif-E3 ubiquitin ligase complex and E2 ubiquitin–conjugating enzymes. The results showed that only the RNA-bound rA3G K128D was efficiently mono- and polyubiquitinated (Fig. 1E). In contrast, the RNA-bound rA3G WT K128 showed barely detectible ubiquitination, and the RNA-free forms of WT K128 or K128D showed practically no ubiquitination (Fig. 1E). These results demonstrate that the interaction between A3G and HIV-1 Vif is D128 dependent and mediated by the RNA copurified with A3G.

### RNA-mediated A3G-Vif interface

**Fig. 2.**
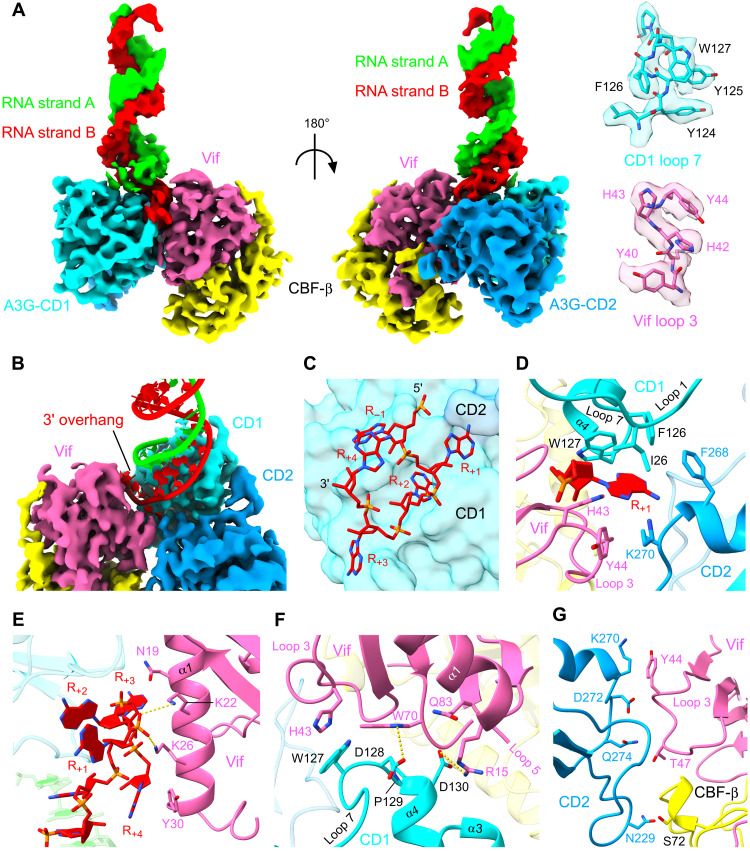
A3G-Vif interface. (**A**) A 3.6-Å cryo-EM structure of the A3G-Vif complex with extended RNA duplex, shown as surface representation. Right: Representative cryo-EM densities of A3G-CD1 and Vif are shown in semitransparency superposed with their atomic models (sticks). (**B**) Close-up view of the RNA branch above the A3G-Vif interface. The cryo-EM density is shown as colored surfaces and RNA atomic model as cylinders/slabs. (**C**) Structure of the 3′ overhang RNA strand (sticks) forming a sharp U turn. Protein structure is shown as surface. (**D**) Detailed interactions around R_+1_ base of the 3′ overhang RNA (sticks/slabs) with amino acids of protein (ribbons/sticks). (**E**) Interactions between 3′ overhang RNA (sticks/slabs) and Vif α1 residues (ribbons/sticks). (**F**) Direct protein interface between A3G-CD1 and Vif. (**G**) Direct protein interface between A3G-CD2 and Vif/CBF-β.

The in vitro–assembled protein complex of rA3G K128D bound to VCBCCR was analyzed by single-particle cryo–electron microscopy (cryo-EM; figs. S1 to S4 and table S1). The population of a protein complex containing A3G and Vif/CBF-β/ELOB/ELOC (VCBC) subcomplex was more dominant in the cryo-EM micrographs than the full complex, likely because of partial dissociation of the CUL5/RBX2 components during the sample vitrification process. The reconstructed 3.6-Å cryo-EM map contained well-resolved α/β domain of Vif, CBF-β, and both domains of A3G (figs. S1 and S2). The cryo-EM map exhibited a density representing a double-stranded RNA of up to 22 base pairs protruding from the A3G-Vif boundary perpendicularly to the Vif-CD1-CD2 plane (Fig. 2A and movie S1). The area with the best local resolution of 2.8 Å coincided with the A3G-Vif interface, where most of the amino acid side chains of the protein subunits and the nucleotides of the RNA strands were confidently built (figs. S2 and S3). Vif binding surface of A3G largely resides in the CD1, which is consistent with previous reports (*6*, *34*). The CD2 shows extra contacts with Vif and CBF-β. In agreement with our biochemical analyses, the structure revealed that A3G and Vif interaction is, in part, mediated by RNA (Fig. 2, A and B). A long protruding A-form RNA duplex is branched above the cavity formed between A3G-CD1 and Vif. While the density of the 5′ end of a branch immediately disappears after the branch point (defined as strand A throughout the text), the 3′ overhang strand (defined as strand B) is intercalated into the cavity formed between A3G-CD1 loop7 and Vif loop3, and the RNA strand exits between CD1 loop1 and Vif α1, forming a sharp U-turn (Fig. 2C). In total, four nucleotides are clearly visible for the 3′ overhang RNA strand where consecutive purine (R)–like base densities are observed. Accordingly, the 3′ overhang RNA strand was modeled as 5′-_+1_RRRR_+4_-3′ (Fig. 2C). Among the four nucleotides, the R_+1_ base plays a pivotal role in the complex assembly anchoring Vif, A3G-CD1, and A3G-CD2. The R_+1_ base fits into a highly hydrophobic pocket composed of H43/Y44 from Vif loop3, I26/F126/W127 from A3G CD1, and F268/K270 from CD2 (Fig. 2D). Adjacent H42 on Vif loop3 forms hydrogen bonds with a ribose-specific 2′-hydroxyl group of R_+1_ ribose and R_−1_-R_+1_ backbone phosphate. Residues of Vif α1 interact with downstream 3′ overhang RNA. K22 and K26 stabilize the sharp kink of the single-stranded RNA (ssRNA) through hydrogen bonds with R_+1_-R_+2_ backbone phosphate. Y30 further supports the curved RNA through a base stacking with R_+4_ base (Fig. 2E).

Direct protein-protein interaction was observed between the A3G-CD1 loop7-α4 patch containing -^128^DPD^130^- motif and Vif. CD1 P129 forms a ring-ring hydrophobic interaction with Vif W70 of loop5. The species-specific Vif sensitivity–determining residue D128 of A3G stabilizes this interaction by forming a hydrogen bond with the N1 amine of the indole ring of W70. D130 forms an additional electrostatic interaction with Vif R15 of α1 (Fig. 2F). R_+1_ base–interacting residues A3G CD1 W127 and CD2 K270 also directly interact with Vif residues H43 and Y44, respectively (Fig. 2, F and G).

In total, eight Vif residues participate in the formation of the complex. Four of them are in direct contact with A3G, with a total 610-Å^2^ buried surface. Six Vif residues are in contact with RNA strand B with 411-Å^2^ buried surface. There is no observed contact between Vif and RNA strand A. Vif H43 and Y44 play dual roles in RNA and A3G recognition, facilitating its unique three-way interaction.

### RNA fork recognition and interdomain configuration of A3G

**Fig. 3.**
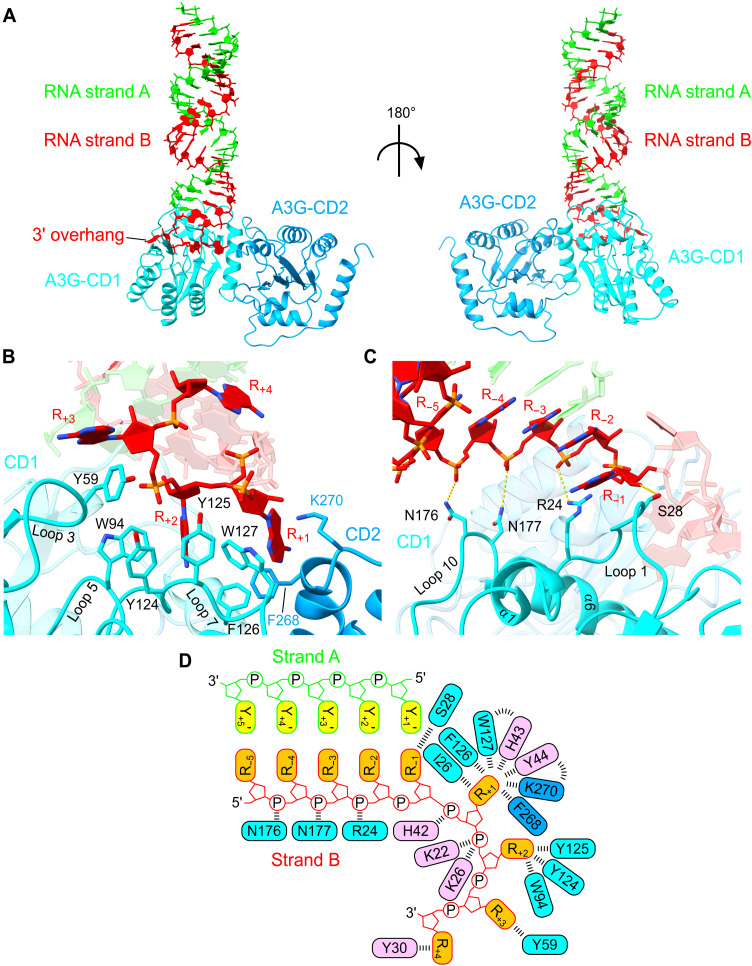
RNA fork branch recognition by A3G and architecture of RNA-mediated A3G dimer. (**A**) Atomic structure of A3G (ribbons) bound to fork RNA (sticks/slabs). (**B**) Interactions between 3′ overhang RNA and A3G residues. (**C**) Interactions between the stem region of the fork RNA and A3G residues. (**D**) Schematic of the fork RNA structure and interactions with CD1, CD2, and Vif residues.

rA3G recognizes the RNA branch through extensive interaction involving loops 1, 3, 5, 7, and 10 (Fig. 3, A to C). Notably, -^124^YYFW^127^- aromatic patch in loop 7 forms a hydrophobic cage to accommodate R_+1_ and R_+2_ bases, which is consistent with its reported role in RNA binding (Fig. 3B) (*6*, *34*). Two additional aromatic residues in CD1, Y59 on loop 3 and W94 on loop 5, form π-stacking with R_+3_ and R_+2_, respectively (Fig. 3B). S28 on loop 1 forms hydrogen bonds with R_−1_ base and a ribose-specific 2′ hydroxyl group of R_−1_. R24 recognizes the R_−3_-R_−2_ backbone phosphate through the major groove. N176 and N177 on loop 10 further hold upstream double-stranded RNA (dsRNA) stem by hydrogen bonds with R_−5_-R_−4_ and R_−4_-R_−3_ backbone phosphates (Fig. 3C). In total, there are 11 residues in CD1 and 2 residues in CD2 participating in the interaction with strand B, while there is no observed interaction with strand A (Fig. 3D). The RNA binding residues and Vif binding residues identified in our structure are all conserved between rA3G and hA3G except for the species-specific residue at position D/K128 (fig. S5A), which further substantiates the use of humanized rA3G as a representative of hA3G. Our structure revealed that conserved F268 and K270 from CD2 also play critical roles in the stable ternary complex formation between A3G, RNA, and Vif-E3 ligase complex. F268A/K270A double mutation in rA3G with K128D displayed severely reduced binding to the VCBCCR complex (fig. S5B). Notably, CD1 loop 8, which has undergone the stability-enhancing loop swapping in our engineered rA3G construct, is not involved in the interface with Vif, CBF-β, or RNA (fig. S5C).

The interdomain configuration of rA3G CD1 and CD2 observed in the complex structure differs from the previously reported apo-form crystal structures of rA3G or soluble variant of hA3G (fig. S6A) (*23*, *24*). In the rA3G apo-form crystal structures, two domains are juxtaposed in a way that the α2 to α4 helix bundles of both domains face the same side. In the structure in complex with Vif E3-ligase, rA3G CD2 undergoes a rotation of nearly 180° around CD1 compared to the apo structures, resulting in the two domains facing opposite directions. Superimposition of Vif/CBF-β–bound A3G and apo form of A3G based on CD1 domain showed steric clashes between CD2 of apo-A3G and CBF-β in the Vif/CBF-β–bound A3G, indicating that the observed CD1-CD2 domain rearrangement is important for Vif binding (fig. S6A). The interdomain interactions are mediated mainly through the contacts between α6 of CD1 and loop 3 α2, which is largely hydrophobic (fig. S6B).

### Architecture of the A3G-Vif-CUL5 E3 ligase complex

**Fig. 4.**
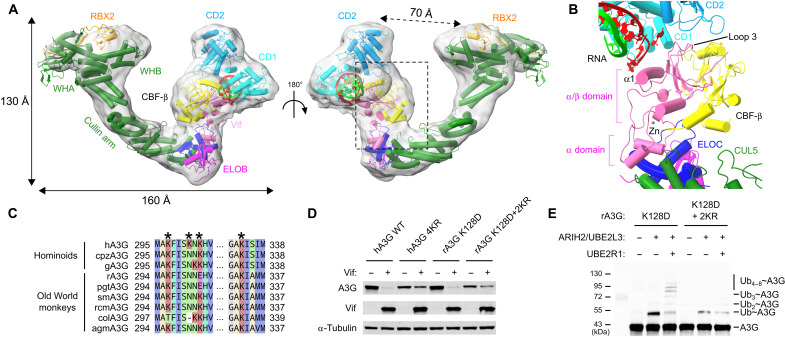
Architecture of the A3G-Vif-CUL5 complex. (**A**) Cryo-EM density map (semitransparent gray, low-pass filtered) of the A3G-VCBCCR complex, superposed with the atomic models (colored by molecules) of the A3G-Vif complex from this study, a subregion of VCBCC complex [Protein Data Bank (PDB) ID: 4N9F], and CUL5/RBX2 (PDB ID: 3DPL, 2ECL, and 2WZK). (**B**) Close-up view of the atomic models in the boxed region in (A), showing protein contacts involving Vif (in pink), RNA (in green and red strands), and other proteins. (**C**) Sequence alignment of primate A3G highlighting target lysine (K) residues for ubiquitination by the Vif-E3 ligase complex. (**D**) Vif-mediated degradation assay of lysine mutants hA3G 4KR (K297/K301/K303/K334 to R) and rA3G 2KR (K296/K333 to R). Western blots show that the A3G level was restored by mutating the target lysine residues for ubiquitination. (**E**) In vitro ubiquitination assay of rA3G lysine 2KR mutant. Monoubiquitinated and polyubiquitinated rA3Gs were reduced by mutating the target lysine residues for ubiquitination.

The full A3G-VCBCCR heptameric complex was reconstructed at 5.4-Å resolution (fig. S4). While the low abundance of particles and the flexibility of the Cullin arm are likely the limiting factors in reaching higher resolution for the A3G-VCBCCR complex, atomic models of each component were fitted into the density map (Fig. 4A). The global architecture of the complex revealed that Vif nucleates the entire complex, simultaneously contacting four host proteins, A3G, CBF-β, ELOC, and CUL5. As was observed for the A3G-VCBC complex, the density corresponding to the double-domain A3G is located next to the α1 loop 3 side of Vif α/β domain and on the opposite side of the α domain where ELOC and CUL5 are bound (Fig. 4B). The elongated Cullin arm of CUL5 extends away from the Vif/CBF-β core, and the curvature of the arm allows the RING domain of RBX2 bound to the CUL5 C-terminal domain to point toward CD2. The distance between the RBX2 RING domain and CD2 is 70 Å (Fig. 4A). This gap is presumably filled with E2 ubiquitin–conjugating enzymes, such as the Ariadne RBR E3 Ubiquitin Protein Ligase 2 (ARIH2)/UBE2L3 pair and UBE2R1 for ubiquitin chain initiation and elongation, respectively (fig. S7, A and B) (*35*–*37*).

In the search for surface primary amine groups for isopeptide bond formation with ubiquitin molecules, two lysine residues, K296 and K333, were salient among six lysines on the rA3G CD2 surface (fig. S7C). K296 and K333 are located 90 and 70 Å away from the zinc finger of RBX2, respectively, where E2 enzymes would bind (fig. S7D). These lysines are equivalent to two of the four primary Vif-targeted lysines identified in hA3G (K297/K301/K303/K334; Fig. 4C) (*36*, *38*). We generated a mutant substituting the potential target lysines on rA3G_K128D_ with arginines (K296R/K333R or 2KR) for Vif-mediated degradation and ubiquitination assays. The 2KR mutant would maintain the positively charged side chains but would block ubiquitination. Similar to the four lysine-to-arginine 4KR mutant hA3G, the 2KR mutant rA3G_K128D_ displayed an elevated level of resistance to Vif-mediated degradation (Fig. 4D), as well as poor ubiquitin chain formation by Vif (Fig. 4E). These results collectively illustrate the overall architecture of the complex and corroborate that the humanized rA3G_K128D_ adopts a spatial domain arrangement highly analogous to that of hA3G when bound to Vif.

### Model of the extended A3G-RNA complex

To gain a whole picture of the duplex RNA stemming from A3G, we performed two-dimensional (2D) classification with a box larger than the optimal box size used for the A3G-VCBC complex that resulted in the 2D class averages with a partial RNA piece (fig. S1, B and C). Accordingly, 2D classes including both ends of the dsRNA helical stalk became more abundant (fig. S4A). The corresponding 3D class shows an extended complex with a unique dumbbell-shaped architecture into which two copies of RNA-bound A3G can be fitted (fig. S4H). The resulting model showed that two A3G protomers bound to each end of the dsRNA helical stalk with approximately 70 Å in length, which accommodates about 24 base pairs (2.2 turns). In this model, strand A for one A3G protomer is equivalent to strand B for the other protomer, resulting in each of the two strands of the dsRNA interacting with one A3G protein (fig. S8A).

How APOBEC3H (A3H), a single-domain APOBEC3 HIV restriction factor, interacts with RNA has been described previously (*39*–*41*). Three crystal structures of A3H from human, chimpanzee, and pig-tailed macaque all display similar protein-RNA interactions where two copies of A3H molecules are bridged by short dsRNA, leaving the two proteins separated by 15 Å. A3G and A3H, both of which show HIV restriction activity, exhibit pronounced differences in their RNA binding mode and the bound RNA structures. First, the fork RNA bound to A3H has one- or two-nucleotides-long 5′ overhang that interacts with A3H and no 3′ overhang, while A3G has four-nucleotides-long 3′ overhang that interacts with A3G and no 5′ overhang. Second, the dsRNA portion in the three structures for A3H all consists of seven base pairs, while A3G has approximately 24 base pairs. Third, A3H contacts both strands of dsRNA at each end, while A3G contacts only one strand at each end (fig. S8, A and B). These differences would highlight a diversified repertoire of RNA recruitment and the resulting HIV restriction mechanisms by APOBEC3 proteins.

## DISCUSSION

The structural model of the A3G-Vif complex has been long sought-after since the identification of A3G as a host factor that restricts HIV infection (*17*). The A3G-Vif interaction has been expected to be purely protein based, as the binary complex structure has been predicted by biochemistry-aided or cross-linking mass spectrometry–aided computational modeling (*29*, *42*). The direct complex formation has been shown for APOBEC3F (A3F) and Vif with the support of a polypeptide linker between A3F and CBF-β (*43*). Our SEC binding analysis and in vitro ubiquitination assay showed that the functional interaction is strictly dependent on RNA copurified with A3G. Our cryo-EM single-particle analysis further revealed that A3G and Vif form an unusual ribonucleoprotein complex in which the 3′ overhang of fork RNA mediates the A3G-Vif interaction. The 3′ overhang ssRNA plays a critical role as “molecular glue” by forming extensive interactions with both proteins. Besides specific interactions, the negatively charged backbone of the 3′ overhang ssRNA plays an important role in gluing together the two highly positively charged surfaces from Vif and A3G-CD1 (fig. S9, A and B).

Among the eight Vif residues involved in the complex formation, K22, K26, and Y30 on α1 and H42 on loop 3, which are previously reported to be A3G binding residues (*29*, *44*, *45*), interact exclusively with RNA. Other adjacent residues H43 and Y44 on loop 3 play critical dual roles in interacting with both RNA and A3G simultaneously. Our structure showed that the Vif-RNA interface area occupies 40% of the interface formed between A3G and Vif. The area around W70 on loop 5 of Vif directly interacts with A3G -^128^DPD^130^-, a previously identified Vif binding motif. The Vif sensitivity–determining residue D128 in the -^128^DPD^130^- motif seems to play a supportive role in stabilizing the A3G P129 and Vif W70 ring-ring interaction. This observation is consistent with the previous report demonstrating that the substitution of the D128 on A3G with a shorter side chain, i.e., D128A, had a minor influence on HIV infectivity in contrast to the near-complete abrogation of infectivity with a long basic side chain substitution D128K (*6*). K128, as possessed by most Old World monkey A3Gs, could destabilize the ring-ring interaction between A3G P129 and Vif W70 because of the potential repulsion between the primary amine group of K128 and N1 of the W70 indole ring. Moreover, a positively charged K128 would be electrostatically disfavored as the surrounding Vif surface area is also positively charged (fig. S9C). Vif is highly variable among lentiviruses, and the amino acid sequence identity between Vif from HIV-1 and SIVmac239, which naturally infects rhesus macaque, is 23%. Notably, HIV-1 Vif residues R15 and K22 are glutamate and histidine in SIVmac239 Vif (fig. S9D). R15/K22 of HIV-1 Vif are both close (~6 Å) to D128 of A3G inside the buried interface (fig. S9E, left). A change to K128 (from D128) in A3G (as in WT rA3G) would generate unfavorable close contact with the same positively charged K22 of Vif (fig. S9E, middle). K128 goes well with a change of HIV-1 Vif residues R15/K22 to R15E/K22H as in SIVmac239 Vif (fig. S9E, right). Except for D/K128, all other A3G residues contacting RNA and Vif are conserved among primate A3G. Thus, the interface between the “humanized” rA3G with HIV-1 Vif-E3 ligase observed here is likely conserved between primate A3G and SIV Vif-E3 ligase complex.

A3G recognizes a unique branch point of fork RNA through extensive interaction with a single strand across the 3′ overhang and duplex regions. The 3′ overhang RNA interacts with several surface-exposed aromatic residues including -^124^YYFW^127^- in loop 7. The RNA in the dsRNA region contacts a series of polar amino acids in loops 1 and 10. It has been reported that A3G binds to cellular and viral RNA with somewhat promiscuous specificity (*46*, *47*). Our results showed that the structured RNA that has been greatly enriched during the purification of the RNA-bound A3G as a single class from the cryo-EM images was classified computationally. The nature of the copurified RNA for A3G is distinctive from that for A3H (*39*–*41*). Therefore, it is possible that rA3G recruits RNA with certain degrees of selectivity for either sequence or secondary structure. It is also likely that our sample preparation specifically favored the enrichment of a certain type of RNA-bound A3G under the experimental conditions. The rA3G-RNA dimer model shows two rA3G binding to both ends of a long dsRNA stem spanning approximately 24 base pairs. The fact that it can be reconstructed to only a low-resolution reconstruction may suggest certain heterogeneity of the dsRNA conformation or sequence with relatively similar lengths, which were enriched through SEC purification. While the biological relevance of this rA3G-RNA dimer is unclear, it is known that the RNA binding of A3G is important for HMM complex assembly in cells (*14*–*16*). As unprotected ssRNA segment in the A3G-bound RNA complex would be degraded by ribonuclease (RNase) in our purification process, the dsRNA-mediated dimer observed here might represent a possible way to dimerize and multimerize on RNA molecules that contain multiple dsRNA stems and ssRNA binding regions for A3G.

**Fig. 5.**
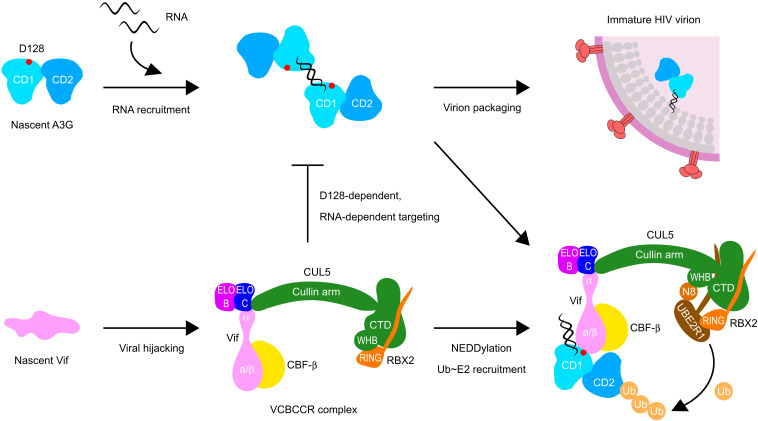
Model of Vif-mediated counteraction of A3G. (**Top**) In HIV-ΔVif infection, newly synthesized A3G first binds to viral genomic RNA or cellular RNAs. Some of these A3G-RNA complexes will further assemble into a large HMM ribonucleoprotein complex. At the same time, some of the initial smaller A3G-RNA complexes can be packaged into a budding HIV virion. These packaged A3Gs then induce lethal hypermutation on HIV reverse transcripts to restrict viral replication. (**Bottom**) On the other hand, Vif, an intrinsically disordered protein, stabilizes itself through binding to CBF-β and CUL5 E3 ubiquitin ligase complex and redirects the E3 ligase complex to target A3G in an RNA-dependent manner to form an A3G-RNA-Vif-E3 ligase complex. After NEDD8 conjugation to CUL5 and recruitment of E2 enzymes thioester-bonded to ubiquitin, A3G is polyubiquitinated, leading to subsequent degradation in the proteasome and inhibition of A3G-RNA packaging into the virion.

Although it is currently unclear why Vif preferentially targets the RNA-bound form of A3G, it is possible that Vif has adapted to specifically target an A3G form that can be a threat to the survival of the viruses. RNA binding–deficient A3G mutants, having one or more mutations in the -^124^YYFW^127^- aromatic patch, are defective in virion packaging and, thus, incompetent in HIV restriction (*6*, *16*, *23*). Therefore, the viruses might have adopted an “avoid overwork” strategy to efficiently reconfigure and exploit the host systems (Fig. 5). Our structure shows that the CD2 zinc-coordinating active site is fully exposed in the complex, and the ssDNA substrates are still accessible to the catalytic pocket. Vif has seemingly opted to target the RNA-bound region of catalytically inactive CD1 instead of directly blocking the active CD2 catalytic pocket.

In summary, this study addresses the long-lasting enigma of the molecular basis of Vif-mediated counteraction with host A3G, central to immune evasion used by HIV and many other lentiviral pathogens. Our structural and functional data shed light on the mechanisms of Vif-A3G coordination and assembly, suggest routes for A3G escape from Vif targeting, and open doors to new avenues for therapeutic development against HIV and other viruses.

## MATERIALS AND METHODS

### Plasmids

Vif from HIV-1 pNL4-3 (GenBank, AF324493.2; residues 1 to 176) with His_6_-tag at N terminus, human ELOB [National Center for Biotechnology Information (NCBI) Reference Sequence, NP_009039.1; residues 1 to 102], human ELOC (NCBI Reference Sequence, NP_001191790.1; residues 17 to 112), and human CBF-β (NCBI Reference Sequence, NP_001746.1; residues 1 to 156) were cloned into each of the four polycistronic cassettes in the pST39 coexpression vector (*48*). Human RBX2 (NCBI Reference Sequence, NP_055060.1; residues 1 to 113) with His_6_-tag at N terminus and CUL5 (NCBI Reference Sequence, NP_003469.2, residues 12 to 780) fused with GB1 at N terminus were cloned into pETDuet-1 coexpression vector. Full-length rA3G (GenBank, AGE34493.1) with stability-enhancing CD1 loop 8 swapping (-^139^CQKRDGPR^146^- to -AEAG-) (*23*, *27*) and E259Q active site mutation (fig. S5A) was cloned into Champion pET SUMO expression vector to express a fusion protein with SUMO at N terminus. For in vitro ubiquitination assay, hemagglutinin (HA)-tag was inserted at the C-terminal end of SUMO-rA3G. For mammalian cell expression, FLAG-hA3G, FLAG-rA3G, HA-hA3G, HA-rA3G, and HIV-1 pNL4-3 Vif were cloned into pcDNA3.1(+) mammalian expression vector. For in vitro ubiquitination assay, human ARIH2, UBE2L3, and UBE2R1 were cloned into pMAL-c5X expression vector to express fusion proteins with maltose binding protein (MBP) at their N terminus. Cloning and mutagenesis were performed with In-Fusion cloning and PrimeSTAR mutagenesis (Clontech) by following the manufacturer’s instructions. The sequences of all the constructs were verified by Sanger DNA sequencing (Genewiz). The multiple sequence alignments were generated with Linneao (https://github.com/beowulfey/linnaeo).

### Vif-mediated degradation assay

One hundred nanograms of FLAG-A3G-pcDNA or HA-A3G-pcDNA variants were cotransfected with 200 ng of either Vif-pcDNA or pcDNA3.1(+) empty vector into human embryonic kidney 293T cells (American Type Culture Collection) in 12-well plates (CELLTREAT) using the X-tremeGENE 9 DNA Transfection Reagent (Roche). Cells were incubated at 37°C in 5.0% CO_2_. At 24 hours after transfection, 2 μM *N*-carbobenzyloxy-l-leucyl-l-leucyl-l-leucinal (Sigma-Aldrich) or dimethyl sulfoxide control was added. At 48 hours after transfection, the cells were washed once with phosphate-buffered saline and lysed in radioimmunoprecipitation assay buffer with 1× cOmplete Protease Inhibitor (Roche). The lysates were then subjected to Western blot with anti-FLAG M2 monoclonal antibody (mAb) from mouse (Sigma-Aldrich), anti–α-tubulin mAb from mouse (GeneTex), and anti-Vif mAb from mouse (National Institutes of Health AIDS Reagent Program #319) as primary antibodies. Cy3-labeled goat anti-mouse mAb (GE Healthcare) was used as a secondary antibody to detect the signal. The fluorescent signal was detected and visualized with Typhoon RGB Biomolecular Imager (GE Healthcare).

### Protein expression and purification

His_6_-Vif/ELOB/ELOC/CBF-β-pST-39 and His_6_-RBX2/GB1-CUL5-pETDuet-1 coexpression vectors were independently transformed into the *Escherichia coli* strain BL21 (DE3). A3G-SUMO-pET expression vector was transformed into the *E. coli* strain Rosetta (DE3). ARIH2-pMAL-c5X, UBE2L3-pMAL-c5X, and UBE2R1-pMAL-c5X expression vectors were transformed into the *E. coli* strain BL21 (DE3). The *E. coli* cells harboring the expression vectors were grown in LB medium at 37°C until the OD_600_ (optical density at 600 nm) reaches 0.6. The recombinant proteins were induced by 0.3 mM isopropyl β-d-1-thiogalactopyranoside at 16°C for 18 hours.

For VCBC complex, the cell pellets were resuspended with buffer A [20 mM tris-HCl (pH 8.0), 500 mM NaCl, and 0.5 mM Tris(2-carboxyethyl)phosphine hydrochloride (TCEP)] containing RNase A (0.1 mg/ml; QIAGEN) and lysed by sonication, and cellular debris was removed by centrifugation. The supernatant containing the His_6_-VCBC complex was loaded onto the Ni-NTA agarose column (QIAGEN). The nickel column was extensively washed with wash buffer [20 mM tris-HCl (pH 8.0), 500 mM NaCl, 50 mM imidazole, and 0.5 mM TCEP], and the protein was eluted with an elution buffer [20 mM tris-HCl (pH 8.0), 500 mM NaCl, 500 mM imidazole, and 0.5 mM TCEP]. The His_6_-tag was cleaved by incubating with PreScission protease overnight. The VCBC complex was subjected to HiLoad 16/600 Superdex 200-pg column (Cytiva) equilibrated with buffer A. The peak fractions were collected and concentrated for mixing with CUL5/RBX2 complex.

For CUL5/RBX2 complex, the cell pellets were resuspended with buffer A and lysed by sonication, and cellular debris was removed by centrifugation. The supernatant containing His_6_-RBX2/GB1-CUL5 was loaded onto the Ni-NTA agarose column. The nickel column was extensively washed with wash buffer [20 mM tris-HCl (pH 8.0), 500 mM NaCl, 50 mM imidazole, and 0.5 mM TCEP], and the protein was eluted with elution buffer [20 mM tris-HCl (pH 8.0), 500 mM NaCl, 500 mM imidazole, and 0.5 mM TCEP]. The His_6_-tag and GB1-tag were cleaved by incubating with PreScission protease overnight. CUL5/RBX2 complex was subjected to Superdex 200 Increase 10/300 GL column (Cytiva) equilibrated with buffer A. The peak fractions were collected and concentrated for mixing with the VCBC complex.

For A3G, the cell pellets were resuspended with buffer B [25 mM Hepes-NaOH (pH 7.5), 500 mM NaCl, and 0.5 mM TCEP] containing RNase A (0.1 mg/ml) and lysed by sonication, and cellular debris was removed by centrifugation. The supernatant containing His_6_-SUMO-A3G was loaded onto Ni-NTA agarose column. The nickel column was extensively washed with wash buffer [25 mM Hepes-NaOH (pH 7.5), 500 mM NaCl, 50 mM imidazole, and 0.5 mM TCEP], and the protein was eluted with elution buffer [25 mM Hepes-NaOH (pH 7.5), 500 mM NaCl, 500 mM imidazole, and 0.5 mM TCEP]. The His_6_-SUMO-tag was cleaved by incubating with PreScission protease overnight. A3G was subjected to Superdex 200 Increase 10/300 GL column equilibrated with buffer B. The peak fractions were concentrated and loaded onto the HiTrap Heparin HP affinity column (Cytiva). The proteins were eluted with a NaCl gradient of 0.25 to 2.0 M. The eluted proteins were further purified using Superdex 200 Increase 10/300 GL column equilibrated with buffer B. The RNA-bound and RNA-free forms were eluted in distinctive peaks and therefore stored separately for the binding test with the VCBCCR complex. The typical A260/280 for RNA-bound form was 1.3, and that for RNA-free form was 0.6.

The VCBCCR complex was formed by mixing the VCBC complex and CUL5/RBX2 by 1:1 molar ratio and incubating on ice for 30 min. The complex was purified using Superdex 200 Increase 10/300 GL column, and the complex fractions were collected and concentrated. The A3G-VCBCCR complex was formed by mixing A3G and VCBCCR by 1:1 molar ratio and incubating on ice for 30 min. The protein complex was further purified using Superdex 200 Increase 10/300 GL column. The peak fraction was isolated and concentrated for cryo-EM work.

For ARIH2, UBE2L3, and UBE2R1, the cell pellets were resuspended with buffer A and lysed by sonication, and cellular debris was removed by centrifugation. The supernatant containing MBP-ARIH2, MBP-UBE2L3, and MBP-UBE2R1 were loaded onto an amylose column (New England Biolabs). The amylose column was extensively washed with wash buffer A, and the protein was eluted with elution buffer [20 mM tris-HCl (pH 8.0), 500 mM NaCl, 20 mM d-maltose, and 0.5 mM TCEP]. The MBP-tag was cleaved by incubating with PreScission protease overnight. Eluted fractions were concentrated and subjected to SEC columns (Superdex 200 Increase 10/300 GL for ARIH2 and Superdex 75 Increase 10/300 GL for UBE2L3 and UBE2R1) equilibrated with buffer A. Peak fractions were collected and concentrated for ubiquitination assay. Protein purity and stoichiometry of the protein complexes were assessed by SDS–polyacrylamide gel electrophoresis (SDS-PAGE) at each purification step.

### SEC binding analysis

Purified A3G and VCBCCR complexes were mixed by 1:1 molar ratio in binding buffer [25 mM Hepes-NaOH (pH7.5), 300 mM NaCl, and 0.5 mM TCEP] and incubated at 4°C for 30 min. The mixture was then subjected to Superdex 200 Increase 10/300 GL column equilibrated with buffer [25 mM Hepes-NaOH (pH7.5), 300 mM NaCl, and 0.5 mM TCEP]. Fractions were concentrated and visualized by SDS-PAGE.

### In vitro ubiquitination assay

Before the ubiquitination assay, Neural precursor cell expressed developmentally down-regulated protein 8 (NEDD8) was conjugated to CUL5 in the VCBCCR complex to activate the E3 ubiquitin ligase for recruiting E2 ubiquitin–conjugating enzymes. The NEDD8 conjugation reaction was performed by mixing 10 μM VCBCCR complex, 0.8 μM E1 NEDD8 activating enzyme (Enzo Life Sciences), 1 μM UBE2F (Enzo Life Sciences), 40 μM NEDD8 (Enzo Life Sciences), inorganic pyrophosphatase from baker’s yeast (15 U/ml) (Sigma-Aldrich), 50 mM tris (pH 7.5), 250 mM NaCl, 5 mM MgCl_2_, 5 mM adenosine triphosphate (ATP), and 1 mM dithiothreitol (DTT) and by incubating for 2.5 hours at room temperature. NEDD8-conjugated VCBCCR (N8 ~ VCBCCR) complex was purified using Superdex 200 Increase 10/300 GL column to remove excess amount of NEDD8 and other enzymes in the reaction.

The mono- and polyubiquitination of A3G were performed by mixing 1 μM HA-A3G (and its variants), 0.4 μM E1 ubiquitin-activating enzyme (Enzo Life Sciences), 0.3 μM ARIH2, 1.8 μM UBE2L3, 3.6 μM UBE2R1 (for polyubiquitination only), 1.5 μM N8~VCBCCR complex, 12 μM ubiquitin (Sigma-Aldrich), inorganic pyrophosphatase from baker’s yeast (15 U/ml; Sigma-Aldrich), 50 mM tris (pH 7.5), 250 mM NaCl, 5 mM MgCl_2_, 5 mM ATP, and 1 mM DTT in 20 μl of reaction volume and by incubating for 2 hours at room temperature. The reaction was stopped by adding 2× sample buffer. The ubiquitinated products were then subjected to Western blot with anti-HA mAb from mouse (Sigma-Aldrich) as a primary antibody. Cy3-labeled goat anti-mouse mAb (GE Healthcare) was used as a secondary antibody to detect the signal. The fluorescent signal was detected and visualized with the Typhoon RGB Biomolecular Imager (GE Healthcare).

### Cryo-EM specimen preparation and data acquisition

The freshly reconstituted protein complex was cross-linked with 1 mM bis-sulfosuccinimidyl suberate (Thermo Fisher Scientific) on ice for 30 min. The reaction was quenched by 50 mM tris (pH 8.0) for an additional 10 min. Four microliters of aliquots of samples at 0.1 to 0.15 mg/ml was applied to graphene oxide–coated Quantifoil R1.2/1.3 gold 400-mesh grids (Electron Microscopy Sciences). The grids were then blotted and vitrified in liquid ethane using Vitrobot Mark IV (Thermo Fisher Scientific). Vitrified grids were screened in Talos F200C (Thermo Fisher Scientific) and Tecnai F20 (FEI) transmission electron microscopes to optimize the freezing conditions.

Cryo-EM data were collected in Glacios (Thermo Fisher Scientific) equipped with Falcon-4 direct electron detector operated at 200 kV in electron counting mode. Movies were collected at a nominal magnification of 150,000× and a pixel size of 0.92 Å in EER format. A total dose of 40 e^−^/Å^2^ per movie was used with a dose rate of 5 to 6 e^−^/Å^2^ per second. A total of 11,803 movies were recorded by automated data acquisition with EPU (Thermo Fisher Scientific).

### Cryo-EM data processing

A total of 11,803 movies were imported into cryoSPARC software package (*49*). The movies were subjected to patch motion correction and contrast transfer function (CTF) estimation in cryoSPARC. The micrographs were manually curated to discard exposures on empty holes or carbon film, which resulted in 11,061 selected micrographs. Reference-free manual particle picking in a small subset of data was performed to generate 2D templates for autopicking. For the A3G-VCBC complex, a total of 5,627,270 particles were picked, extracted with a box size of 320 pixels, and binned by a factor of 4, on which iterative rounds of 2D classification were performed. To generate well-defined ab initio volumes, 795,392 particles from 2D classes with clear internal features were reextracted without binning. 3D ab initio reconstruction was then performed to generate six classes. Because particles with rare views are often classified into less well-defined classes or “junk” classes during 2D classification, we salvaged those good particles with rare views from a wider selection of 2D classes. A total of 1,806,858 particles from an intermediate round of 2D classification with distinct shape were used in the heterogeneous refinement with six ab initio classes. To sort out particles with high-resolution features from junk particles, we performed heterogeneous refinement by using a single copy, duplicate, and triplicate of the six ab initio classes as starting volumes. A similar approach has been reported in (*50*). The best particle stack is derived from the top three classes including a single class with distinctive four blobs (CD1, CD2, Vif/CBF-β, and ELOB/C) and two classes with a long helical feature of dsRNA. These classes were yielded in the heterogeneous refinement with triplicates of the ab initio volumes, and they were used for the subsequent 3D refinement jobs. Nonuniform refinement (*51*) was performed to yield a 4.0-Å resolution map. After optimizing dynamic mask and performing global CTF refinement, a 3.6-Å resolution final map was obtained and used for atomic modeling.

For the A3G-RNA dimer complex and A3G-VCBCC complex, a total of 5,032,127 particles were picked, extracted with a box size of 512 pixels, and binned by a factor of 4, on which iterative rounds of 2D classification was performed. For the A3G-RNA dimer, 1,636,131 particles from 2D classes with clear internal features were reextracted without binning. 3D ab initio reconstruction was then performed to generate four classes. A single class containing 449,679 particles showing a feature of dsRNA stalk with two blobs, one at each end of the stalk, was selected. Two rounds of heterogeneous refinement were performed to obtain a more homogeneous particle stack, which includes 116,460 particles. This particle stack was used for nonuniform refinement, which yielded the final 4.5-Å resolution map. For the A3G-VCBCCR complex, more extensive classification was needed to separate classes with CUL5/RBX2 from the A3G-VCBC complex. A total of 72,559 particles from the 2D classification were reextracted without binning. 3D ab initio reconstruction and 3D classification were then performed to generate two classes. A class from 38,571 particles showing a feature of extended Cullin arm was selected and used for nonuniform refinement, which yielded the final 5.4-Å resolution map. All resolution evaluation was performed on the basis of the gold-standard criterion of Fourier shell correlation (FSC) coefficient at the 0.143 cutoff (*52*).

### Model building and refinement

Atomic models derived from crystal structures of apo-rA3G [Protein Data Bank (PDB) ID: 6P3X] and VCBCC complex (PDB ID: 4N9F) were docked into the cryo-EM maps using UCSF Chimera (*53*). For A3G, because of the obvious difference in interdomain orientation, the apo-rA3G structure was first split into CD1 and CD2 at R194-H195 of the interdomain linker region, and the two domains were independently docked into the cryo-EM map. α Domain of Vif (residues D113 to I160), ELOB, and ELOC were visible only at low isosurface threshold, therefore truncated from the model. The model was refined with the phenix.real_space_refine module in Phenix, with secondary structure restraints and geometry restraints (*54*, *55*). We then manually adjusted the protein side-chain conformation and, when necessary, moved the main chains to match the density map using COOT (*56*). This allowed us to identify extra densities for RNA. Well-defined nucleotide densities, along with the base pairs, facilitated the RNA model building process. The map resolution of the 3′ overhang RNA (R_+1_-R_+4_ in strand B) is sufficient to distinguish between purine and pyrimidine, while the map resolution of the dsRNA helix is insufficient for de novo atomic modeling; therefore, we traced the main chain using COOT. The atomic models went through cycles of real-space refinement in Phenix (*57*). The final atomic models were validated using the comprehensive cryo-EM validation tool implemented in Phenix (table S1) (*57*). All structural figures were generated with UCSF Chimera or ChimeraX (*58*).

## Supplementary Materials

### This PDF file includes:

Figs. S1 to S9 Table S1 References

## Notes

View/request a protocol for this paper from *Bio-protocol*.
