# Supplementary material for: Structural basis for HIV-1 antagonism of host APOBEC3G via Cullin E3 ligase
Source: Sci Adv. 2023 Jan 4;9(1):eade3168. doi: 10.1126/sciadv.ade3168 (PMC9812381; doi:10.1126/sciadv.ade3168)
Supplement: Supplementary file 1 — Figs. S1 to S9 Table S1 References [file sciadv.ade3168_sm.pdf]

Science Advances
AAAS

Supplementary Materials for

Structural basis for HIV-1 antagonism of host APOBEC3G via Cullin E3 ligase

Fumiaki Ito *et al.*

Corresponding author: Z. Hong Zhou, hong.zhou@ucla.edu; Xiaojiang S. Chen, xiaojiac@usc.edu

Sci. Adv. **9**, eade3168 (2022)
DOI: 10.1126/sciadv.ade3168

The PDF file includes:

Figs. S1 to S9
Table S1
Legend for movie S1
References

Other Supplementary Material for this manuscript includes the following:

Movie S1

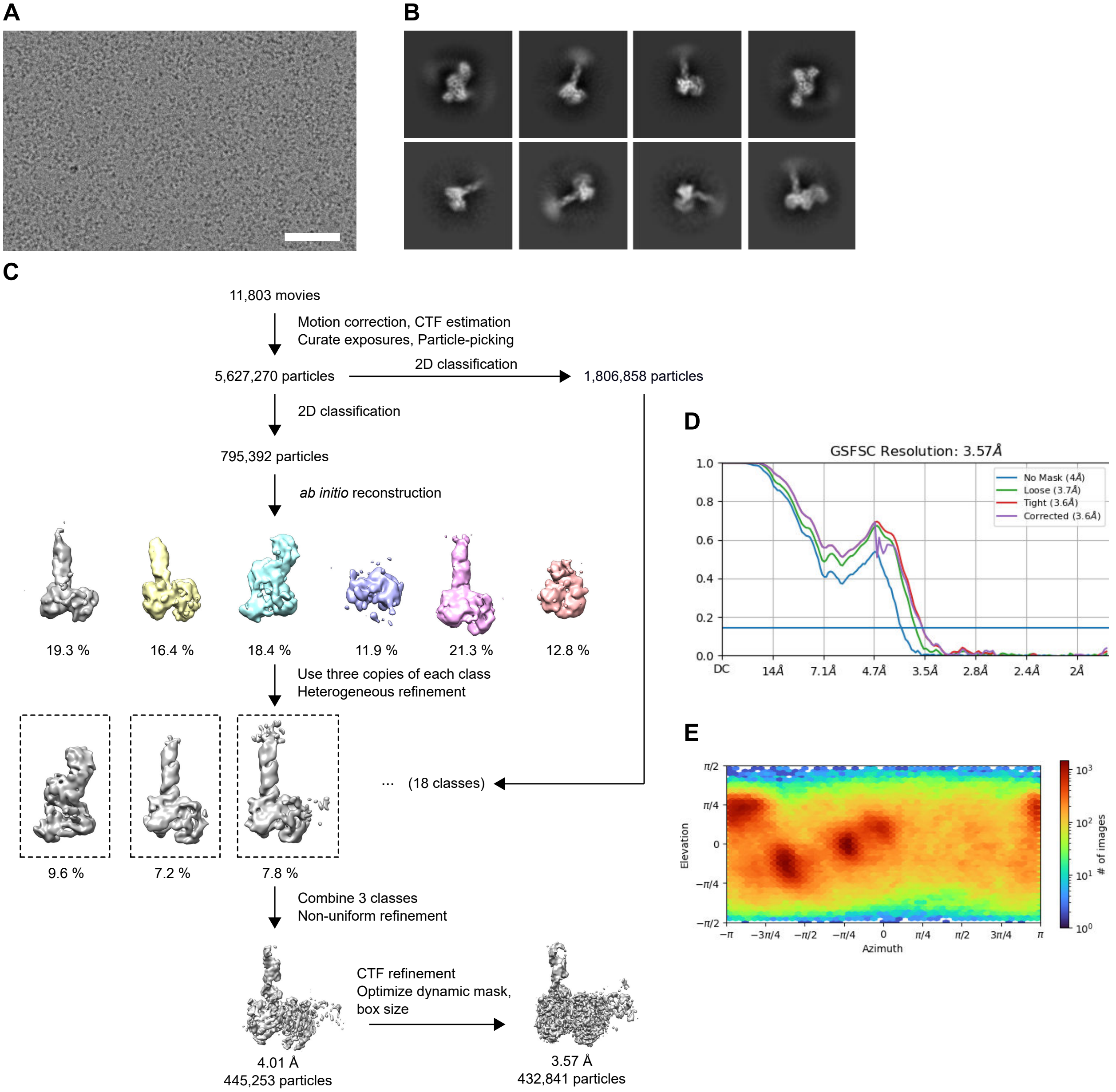

Fig. S1. Imaging, reconstruction, and resolution evaluation of the A3G-VCBC complex. (**A**) Representative cryo-EM raw image of the A3G-VCBC complex. Scale bars: 50 nm. (**B**) Representative 2D class averages of the A3G-VCBC complex. (**C**) Cryo-EM image processing workflow of the A3G-VCBC complex. (**D**) Global resolution estimation of the A3G-VCBC complex based on the "gold standard" Fourier shell correlation (FSC) coefficient of 0.143 criterion. (**E**) Angular distribution plot of the particles containing the A3G-VCBC complex.

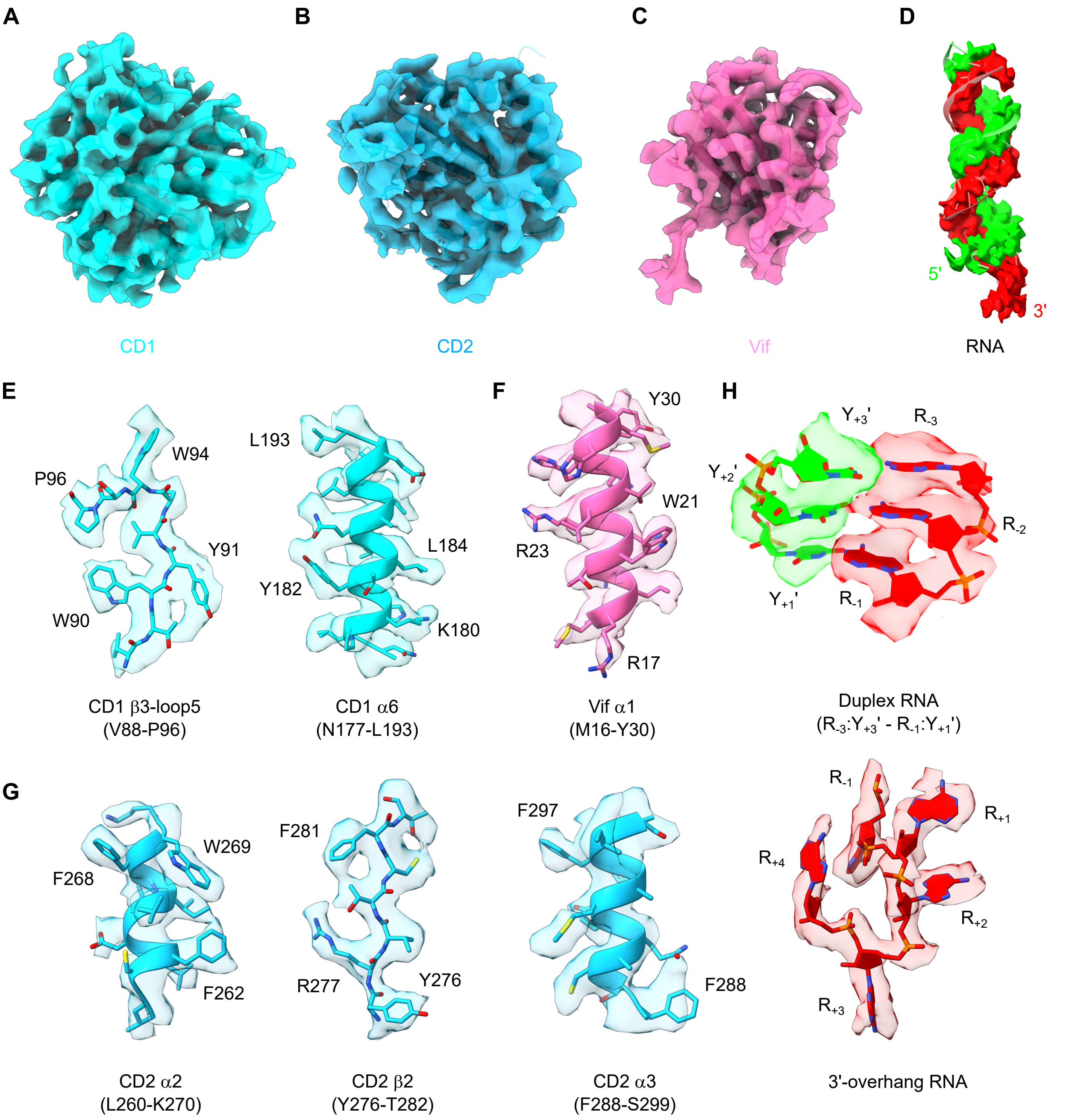

Fig. S2. Cryo-EM density maps of the protein and RNA components. Segmented cryo-EM densities of (**A**) A3G-CD1, (**B**) A3G-CD2, (**C**) Vif, and (**D**) RNA and representative local regions of (**E**) A3G-CD1, (**F**) Vif, (**G**) A3G-CD2, and (**H**) RNA, superposed with atomic models of amino acid side chains and nucleotide bases (ribbons and sticks). The densities are displayed with contour levels of 0.16-0.18.

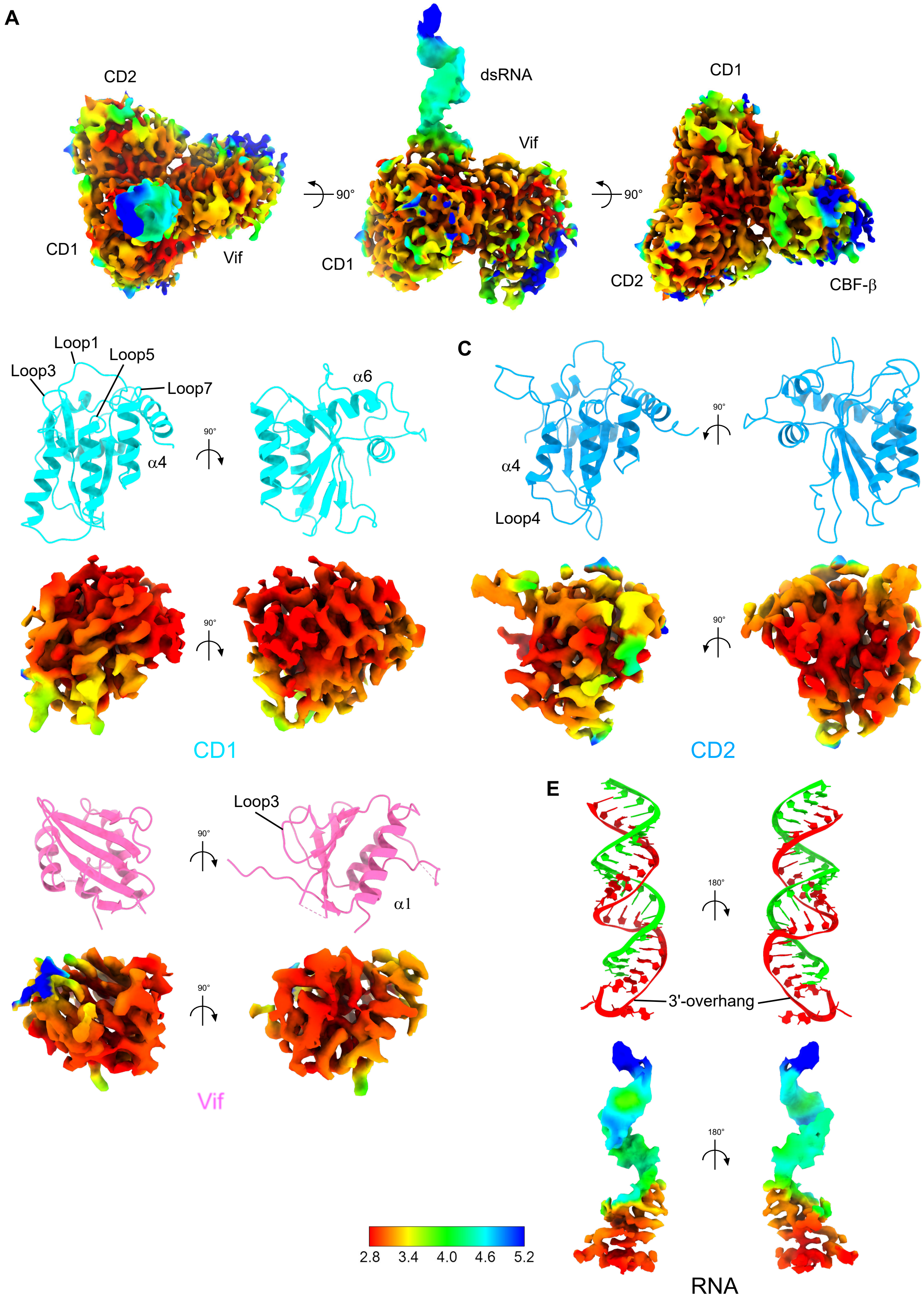
A
CD2
dsRNA
Vif
CD1
90°
CD1
Vif
CD1
90°
CD2
CBF-β
B
Loop1
Loop5
Loop3
Loop7
α6
α4
90°
90°
CD1
C
90°
α4
Loop4
90°
CD2
D
Loop3
90°
α1
90°
Vif
E
180°
3'-overhang
180°
2.8
3.4
4.0
4.6
5.2
RNA

Fig. S3. Local resolution evaluation of the A3G-VCBC complex. Local resolution distribution of (**A**) overall density, (**B**) A3G-CD1, (**C**) A3G-CD2, (**D**) Vif, and (**E**) RNA. The densities are displayed with contour levels of 0.24 for the proteins and 0.20 for RNA.

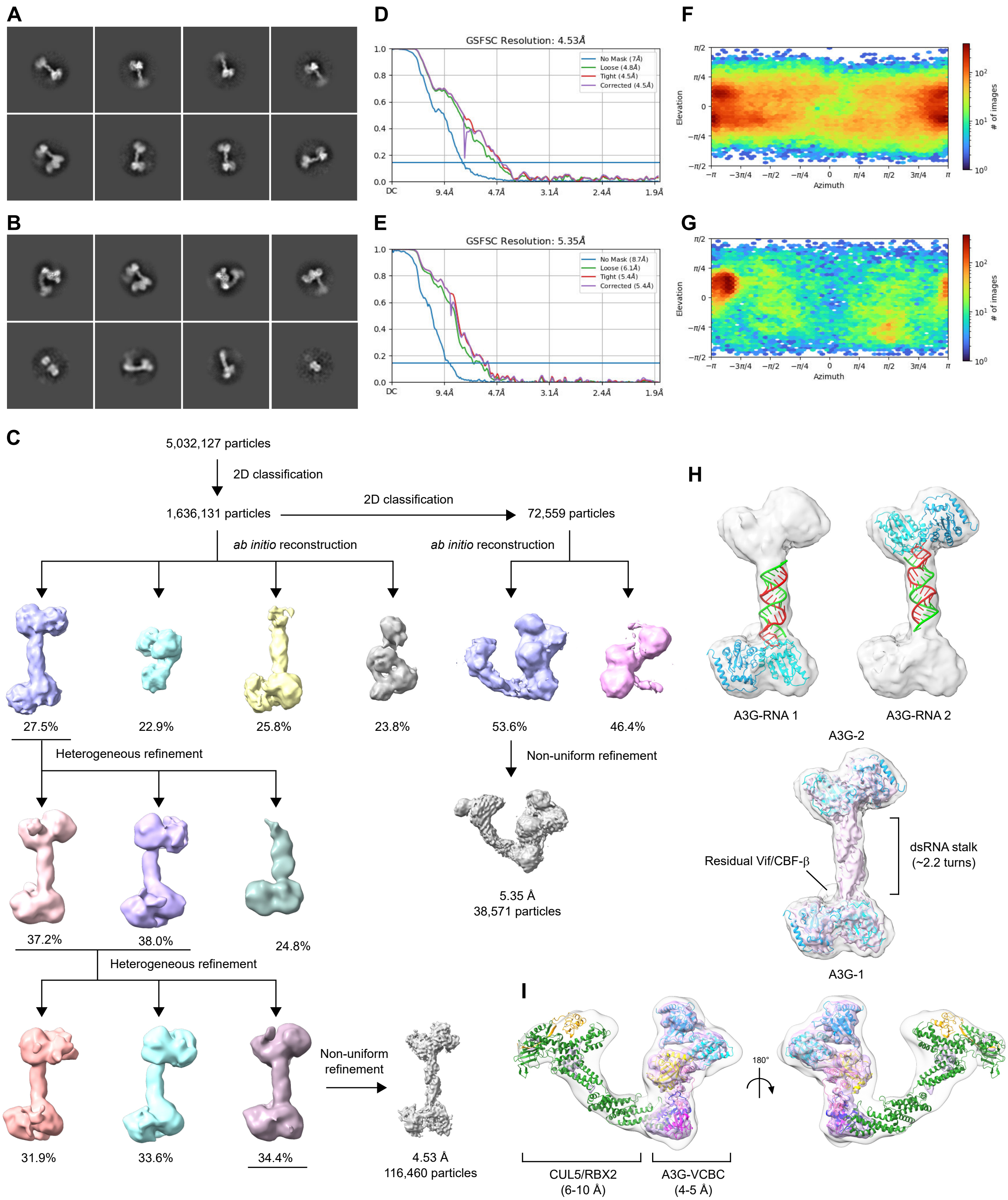

A
B
C
D
GSFSC Resolution: 4.53Å
No Mask (7Å)
Loose (4.8Å)
Tight (4.5Å)
Corrected (4.5Å)
E
GSFSC Resolution: 5.35Å
No Mask (8.7Å)
Loose (6.1Å)
Tight (5.4Å)
Corrected (5.4Å)
F
G
Elevation
Azimuth
# of images
5,032,127 particles
2D classification
1,636,131 particles
2D classification
72,559 particles
ab initio reconstruction
ab initio reconstruction
27.5%
22.9%
25.8%
23.8%
53.6%
46.4%
Heterogeneous refinement
Non-uniform refinement
37.2%
38.0%
24.8%
5.35 Å
38,571 particles
Heterogeneous refinement
31.9%
33.6%
34.4%
Non-uniform refinement
4.53 Å
116,460 particles
H
A3G-RNA 1
A3G-RNA 2
A3G-2
Residual Vif/CBF-β
dsRNA stalk (~2.2 turns)
A3G-1
I
180°
CUL5/RBX2 (6-10 Å)
A3G-VCBC (4-5 Å)

Fig. S4. Cryo-EM reconstruction of the A3G-RNA dimer and the A3G-VCBCCR complex. Representative 2D class averages of (**A**) the A3G-RNA dimer and (**B**) the A3G-VCBCCR complex. (**C**) Cryo-EM image processing workflow of the A3G-RNA dimer and the A3G-VCBCCR complex. The global resolution estimation (based on the Fourier shell correlation (FSC) 0.143 criterion) of (**D**) the A3G-RNA dimer and (**E**) the A3G-VCBCCR complex. Angular distribution plot of the particles containing (**F**) the A3G-RNA dimer and (**G**) the A3G-VCBCCR complex in the final reconstruction. (**H**) Atomic model fitting of the A3G-RNA dimer. Two copies of the A3G-RNA complex model were individually docked into the density map (top). A sharpened high-threshold map was overlaid into the unsharpened low-threshold map to illustrate the helical feature of the dsRNA connecting the two A3G (bottom) (**I**) Atomic model fitting of A3G-VCBCCR complex. A sharpened high-threshold map was overlaid into the unsharpened low-threshold map to illustrate the secondary structure features of A3G-VCBC subregion.

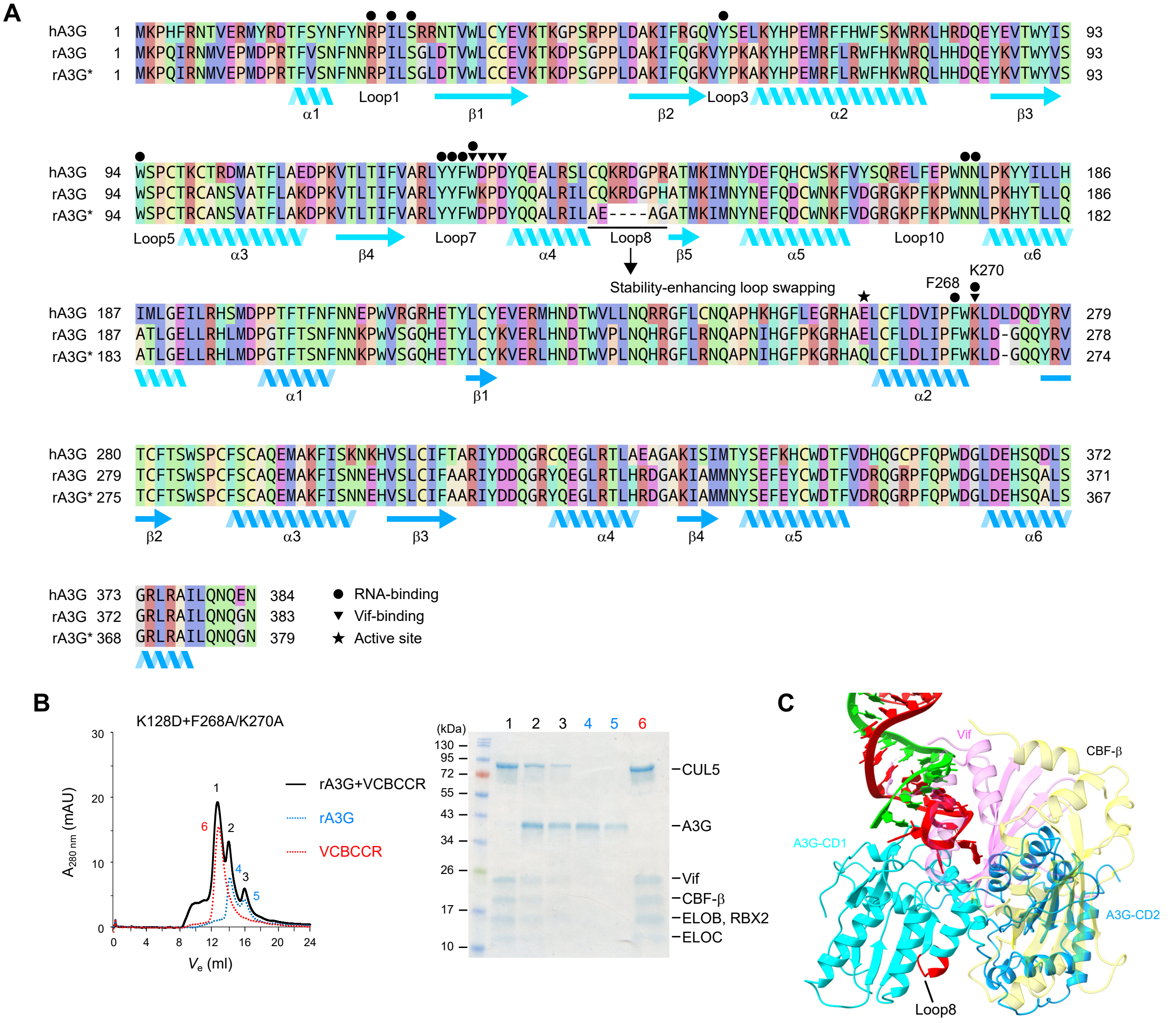

Fig. S5. Construct design and functionally important residues of A3G. (**A**) Amino acid sequence alignment of hA3G and rA3G. The engineered rA3G used in this study is included as rA3G*. RNA-binding residues (marked with circles) and Vif-binding residues (marked with triangles) are all conserved in hA3G and rA3G except for the species-specific residue D/K at position 128 on loop7 of CD1. (**B**) Binding analysis of rA3G K128D+F268A/K270A (RNAbound form) and VCBCCR complex by SEC. Addition of CD2 mutations in rA3G caused no major peak shift when mixed with VCBCCR complex. SDS-PAGE image shows the protein components in the indicated SEC fractions. (**C**) Location of CD1 loop8 in the A3G-VCBC complex showing the chimeric loop is exposed to solvent and not in contact with Vif or RNA.

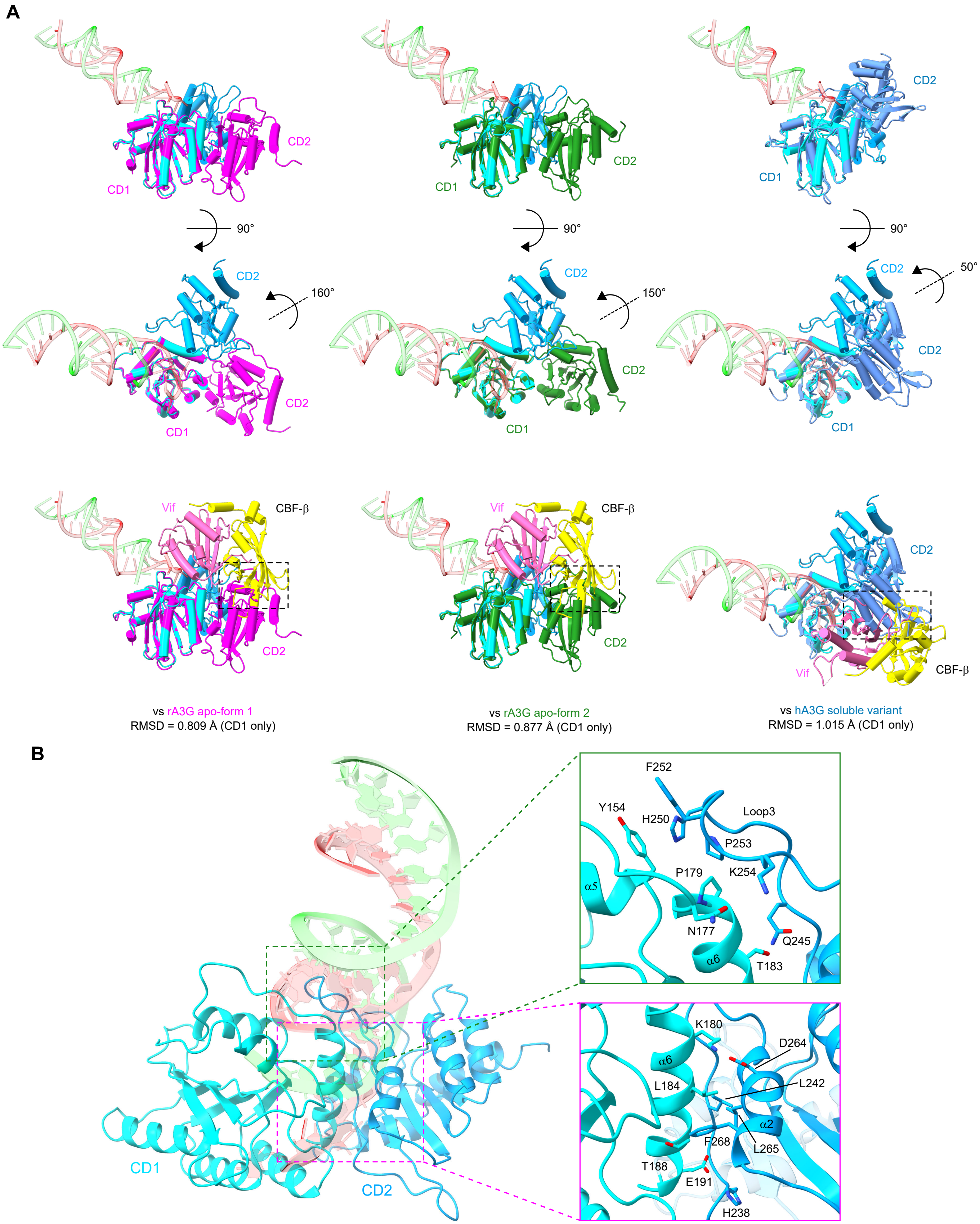
A
CD2
CD1
CD2
CD1
CD2
CD1
90°
90°
90°
CD2
160°
CD2
150°
CD2
50°
CD2
CD2
CD2
CD1
CD1
CD1
Vif
CBF-β
Vif
CBF-β
CD2
CD2
CD2
CD2
Vif
CBF-β
vs rA3G apo-form 1
RMSD = 0.809 Å (CD1 only)
vs rA3G apo-form 2
RMSD = 0.877 Å (CD1 only)
vs hA3G soluble variant
RMSD = 1.015 Å (CD1 only)
B
F252
Y154
H250
Loop3
P253
P179
K254
α5
N177
Q245
α6
T183
K180
D264
α6
L242
L184
α2
F268
L265
T188
E191
H238
CD1
CD2

Fig. S6. Inter-domain interface and orientation of A3G in the complex with Vif and the comparison with apo-form A3G structures. (**A**) Comparison of the inter-domain orientations of rA3G (in cyan) in the rA3G-Vif complex with two apo-forms of rA3G (PDB ID: 6P3X colored in magenta in the left, PDB ID: 6P40 colored in green in the middle) (*23*) and hA3G soluble variant (PDB ID: 6WMA colored in light-blue) (*24*). Top two rows are orthogonal views of A3G structures superimposed over CD1 highlighting the variation of CD2 positions. The images shown in the bottom row are the superimposition of Vif/CBF-β-bound A3G and apo-A3G structures. Steric clashes are indicated by dashed boxes. (**B**) Inter-domain interface observed in the A3G-Vif complex structure. Close-up views depict CD1 α5-α6 patch and CD2 loop3 interface (top), and CD1 α6 and CD2 α2 interface (bottom).

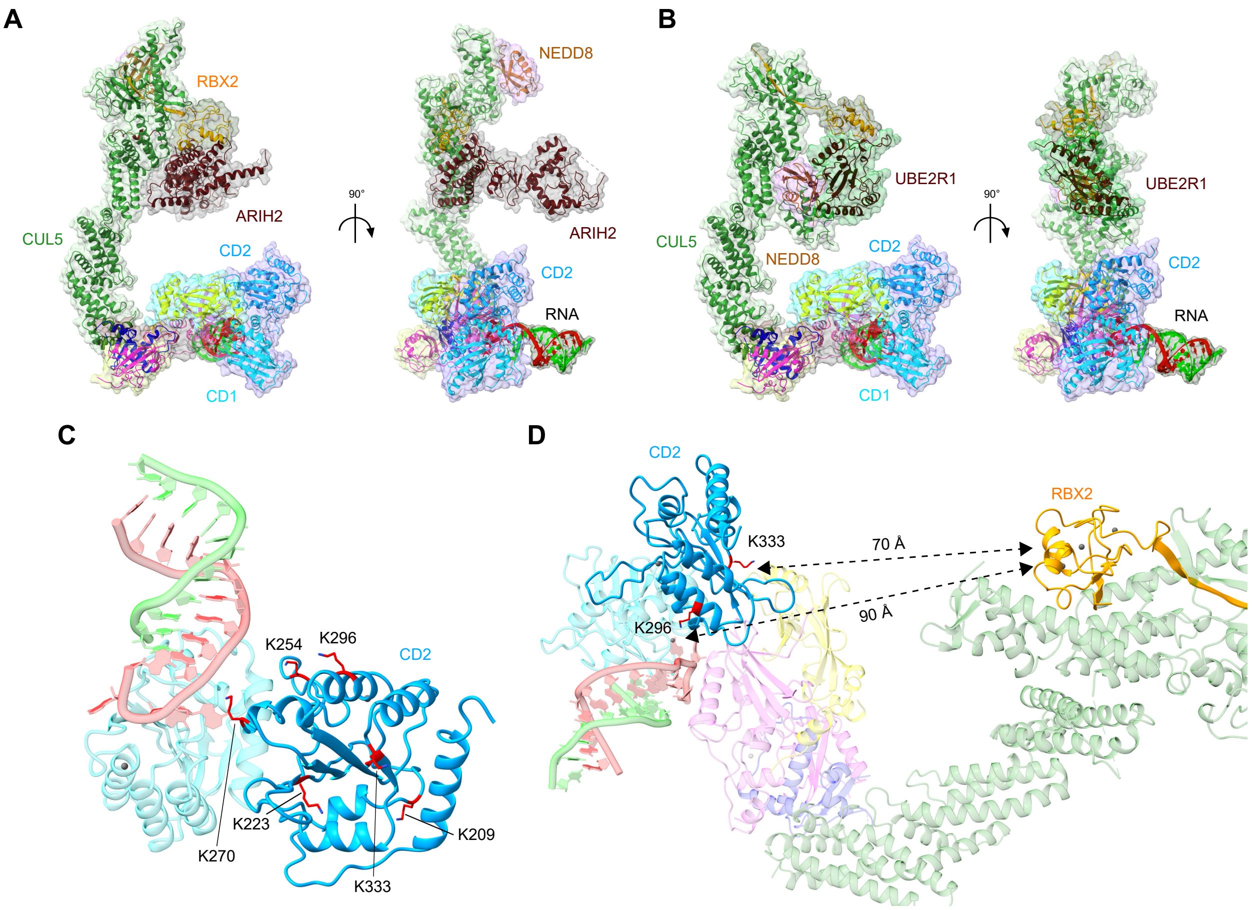

Fig. S7. Extended model of A3G-Vif complex for ubiquitin transfer and target ubiquitination sites on A3G-CD2. (**A**) ARIH2-bound model (PDB ID: 7ONI) (*37*) of the A3G-Vif complex for mono-ubiquitination of A3G. (**B**) UBE2R1-bound model (PDB ID: 6NYO, 6TTU) (*59, 60*) of the A3G-Vif complex for poly-ubiquitination. (**C**) Surface lysine residue mapping on CD2. (**D**) Distance between RING domain of RBX2 and K296 and K333 in CD2.

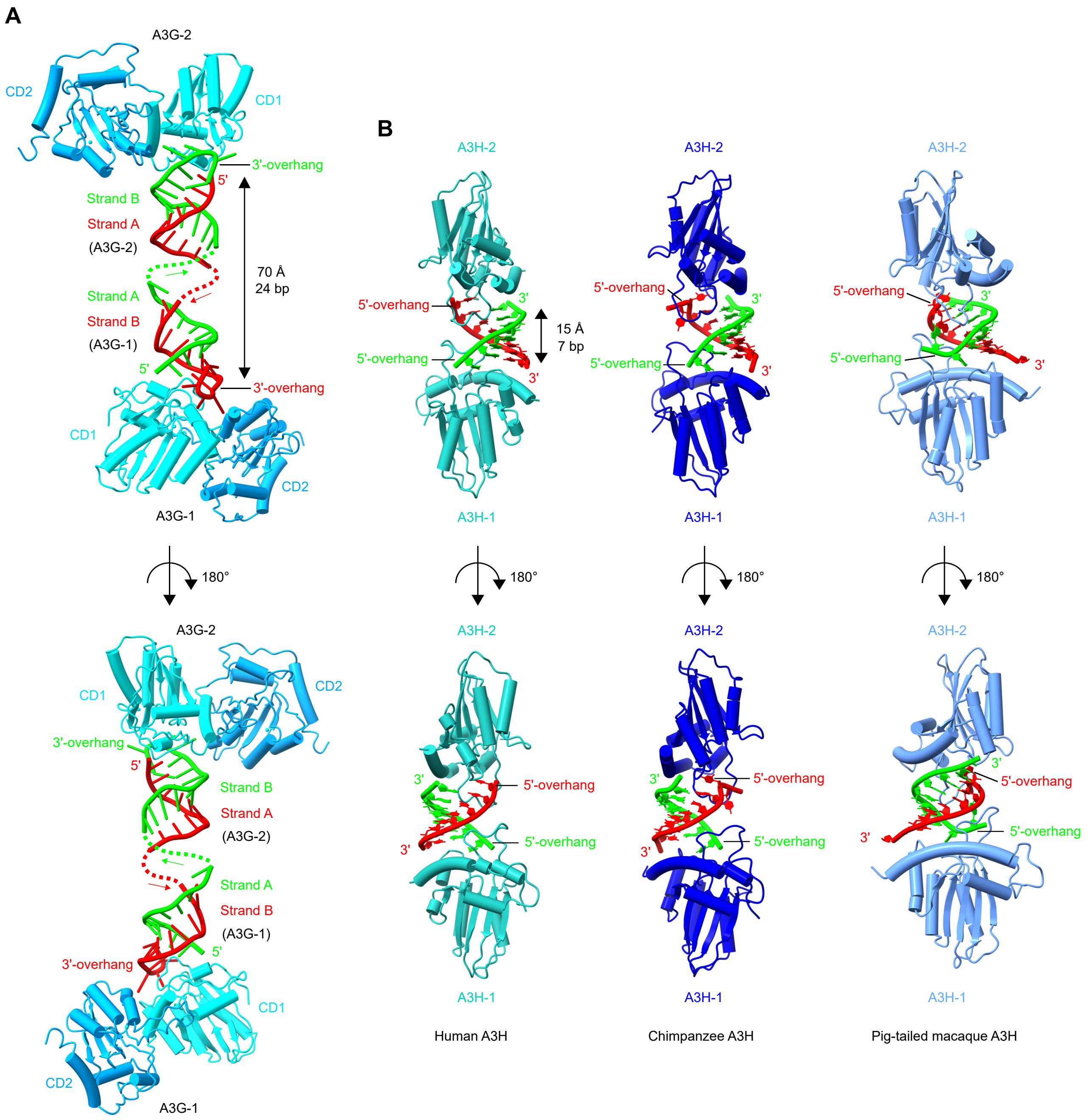

Fig. S8. Comparison of RNA-mediated dimer of A3G and A3H. (**A**) Model of an RNA-mediated dimer of A3G. Predicted paths for the dsRNA are depicted in dash lines. (**B**) Structures of the RNA-mediated dimer of A3H from human (PDB ID: 6B0B) (*40*), chimpanzee (PDB ID: 5Z98) (*41*), and pig-tailed macaque (PDB ID: 5W3V) (*39*).

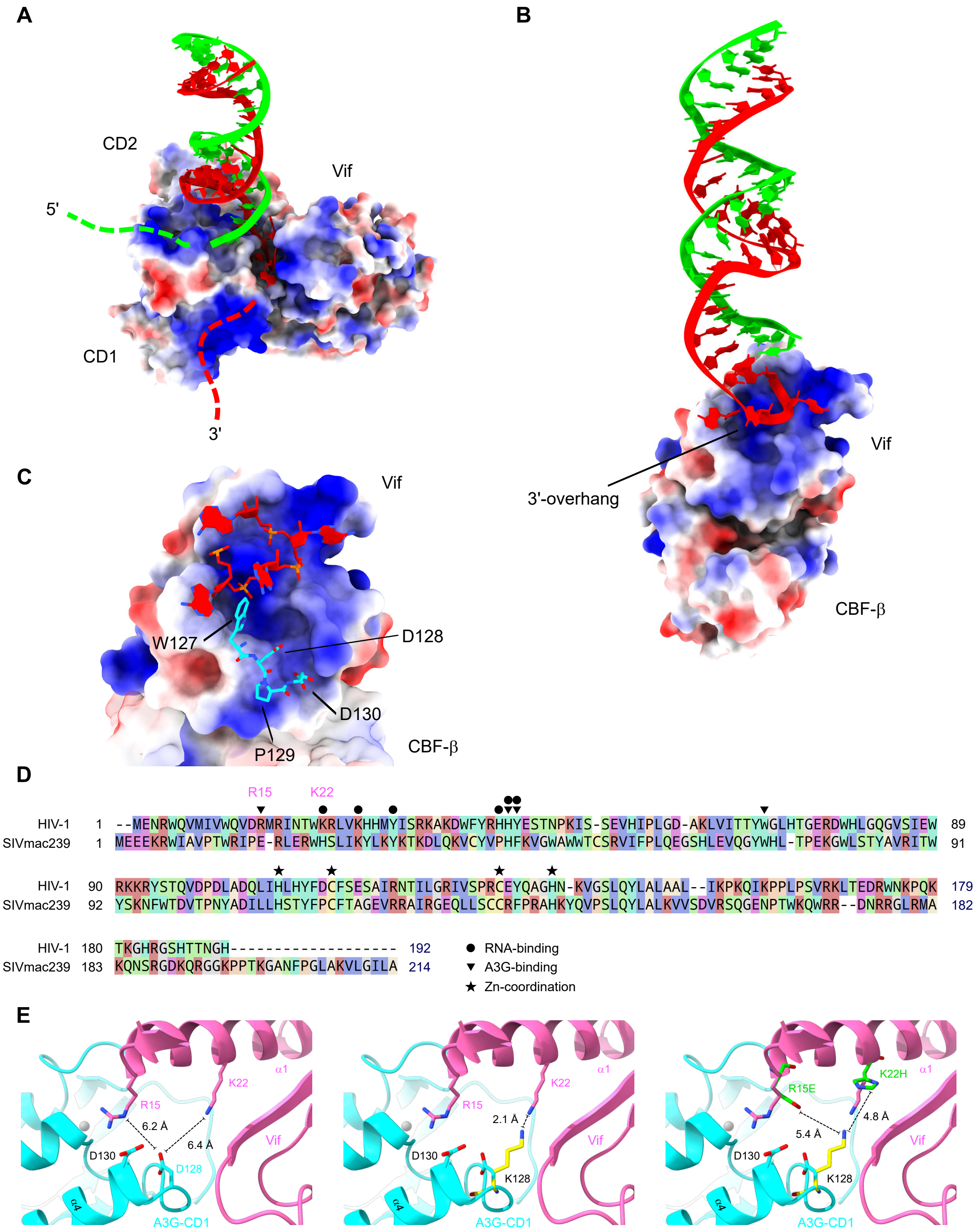

A
CD2
Vif
5'
CD1
3'
B
Vif
3'-overhang
CBF-β
C
Vif
W127
D128
D130
P129
CBF-β
D
R15
K22
HIV-1 1 --MENRWQVMIVWQVDRMRINTWKRLVKHHMYISRKAKDWFYRHHYESTNPKIS-SEVHIPLGD-AKLVITTYWGLHTGERDWHLGQGVSIEW 89
SIVmac239 1 MEEEKRWIAVPTWRIPE-RLERWHSLIKYLKYKTKDLQKVCYVPHFKVGWAWWTCSRVIFPLQEGSHLEVQGYWHL-TPEKGWLSTYAVRITW 91
HIV-1 90 RKKRYSTQVDPDLADQLIHLHYFDCFSESAIRNTILGRIVSPRCEYQAGHN-KVGSLQYLALAAL--IKPKQIKPPLPSVRKLTEDRWNKPQK 179
SIVmac239 92 YSKNFWTDVTPNYADILLHSTYFPCFTAGEVRRAIRGEQLLSCCRFPRAHKYQVPSLQYLALKVVSDVRSQGENPTWKQWRR--DNRRGLRMA 182
HIV-1 180 TKGHRGSHTTNGH------------------- 192
SIVmac239 183 KQNSRGDKQRGGKPPTKGANFPGLAKVLGILA 214
● RNA-binding
▼ A3G-binding
★ Zn-coordination
E
R15
K22
α1
6.2 Å
6.4 Å
D130
D128
Vif
α4
A3G-CD1
R15
K22
α1
2.1 Å
D130
K128
Vif
α4
A3G-CD1
R15E
K22H
α1
5.4 Å
4.8 Å
D130
K128
Vif
α4
A3G-CD1

Fig. S9. Surface electrostatic potential of A3G and Vif, and the insights into SIV Vif-rA3G interaction. (**A**) Electrostatic potential of the A3G-Vif complex around the RNA-binding regions. Positively charged surfaces of A3G and Vif are located next to each other across the cleft occupied with 3′-overhang RNA. Hypothetical paths for the extended 5′-overhang and 3′-overhang are depicted in dashed lines. (**B**) Electrostatic potential of RNA-binding region of Vif, highlighting a highly positively charged surface that contacts 3′-overhang RNA. (**C**) The positively charged surface of Vif extends to the A3G-binding region that contacts the -^{128}DPD^{130}- motif in A3G. The surface area of A3G and Vif/CBF-β is colored according to the calculated electrostatic potential from -10.0 kT/e (red) to +10.0 kT/e (blue). (**D**) Sequence alignment of Vif from HIV-1 and SIVmac239. RNA-binding residues and A3G-binding residues identified in HIV-1 Vif are marked with circles and triangles, respectively. Conserved zinc-coordinating residues are marked with stars. HIV-1 R15 and K22 are glutamate and histidine, respectively in SIVmac239. These two residues are at the interface with the A3G -^{128}DPD^{130}-motif and may play an important role in differentiating hA3G and rA3G. (**E**) Insights into the interaction between rA3G and SIVmac239. The A3G D128 is ~6.4 Å away from K22 of HIV-1 Vif while A3G D130 interacts with Vif R15 (left). When A3G D128 is changed to lysine (K128, in yellow stick) as in rA3G, K128 would have an unfavorable repulsive close contact with Vif K22 (middle). K22 is histidine in SIVmac239 Vif (E22H, in green stick), which creates space for K128 to extend towards Vif. HIV-1 Vif R15 is glutamate in SIVmac239 Vif (R15E, in green stick), which can adopt an electrostatically favored rotamer conformation with K128 (right).

Table S1. Cryo-EM data collection, refinement, and validation statistics

	A3G-VCBC	A3G-VCBCCR	A3G-RNA dimer
**Data collection**			
Magnification	150,000	150,000	150,000
Voltage (keV)	200	200	200
Electron exposure ($e^-/Å^2$)	40	40	40
Defocus range (μm)	-1.2 to -3.0	-1.2 to -3.0	-1.2 to -3.0
Pixel size (Å)	0.92	0.92	0.92
Symmetry imposed	C1	C1	C1
Initial particle images	5,627,270	5,032,127	5,032,127
Final particle images	432,841	38,571	116,460
Map resolution (Å)	3.57	5.35	4.53
FSC threshold	0.143	0.143	0.143
Map resolution range (Å)	2.6-5.8	4.0-10.0	3.8-9.0
**Refinement**			
Initial model used (PDB)	4N9F, 6P3X		
Model resolution (Å)	3.9		
FSC threshold	0.5		
Map sharpening B factor ($Å^2$)	-142.3		
No. non-hydrogen atoms	6297		
Protein residues	636		
Nucleotides	47		
Ligands	2 Zn		
B-factors			
Protein	31.22		
Nucleotide	95.68		
Ligand	67.55		
R.m.s. deviations			
Bond lengths (Å)	0.003		
Bond angles (°)	0.597		
Validation			
MolProbity score	2.00		
Clash score	13.10		
Poor rotamers (%)	0.53		
Ramachandran plot			
Favored (%)	94.57		
Allowed (%)	5.43		
Disallowed (%)	0.00		

Movie S1.
Cryo-EM structure of A3G-Vif complex, highlighting the RNA-mediated A3G-Vif interactions

REFERENCES AND NOTES

1. B. Mangeat, P. Turelli, G. Caron, M. Friedli, L. Perrin, D. Trono, Broad antiretroviral defence by human APOBEC3G through lethal editing of nascent reverse transcripts. *Nature* **424**, 99–103 (2003).

2. R. S. Harris, K. N. Bishop, A. M. Sheehy, H. M. Craig, S. K. Petersen-Mahrt, I. N. Watt, M. S. Neuberger, M. H. Malim, DNA deamination mediates innate immunity to retroviral infection. *Cell* **113**, 803–809 (2003).

3. H. Zhang, B. Yang, R. J. Pomerantz, C. Zhang, S. C. Arunachalam, L. Gao, The cytidine deaminase CEM15 induces hypermutation in newly synthesized HIV-1 DNA. *Nature* **424**, 94–98 (2003).

4. D. Lecossier, F. Bouchonnet, F. Clavel, A. J. Hance, Hypermutation of HIV-1 DNA in the absence of the Vif protein. *Science* **300**, 1112 (2003).

5. V. Zennou, D. Perez-Caballero, H. Gottlinger, P. D. Bieniasz, APOBEC3G incorporation into human immunodeficiency virus type 1 particles. *J. Virol.* **78**, 12058–12061 (2004).

6. H. Huthoff, M. H. Malim, Identification of amino acid residues in APOBEC3G required for regulation by human immunodeficiency virus type 1 Vif and virion encapsidation. *J. Virol.* **81**, 3807–3815 (2007).

7. G. Haché, M. T. Liddament, R. S. Harris, The retroviral hypermutation specificity of APOBEC3F and APOBEC3G is governed by the C-terminal DNA cytosine deaminase domain. *J. Biol. Chem.* **280**, 10920–10924 (2005).

8. E. N. C. Newman, R. K. Holmes, H. M. Craig, K. C. Klein, J. R. Lingappa, M. H. Malim, A. M. Sheehy, Antiviral function of APOBEC3G can be dissociated from cytidine deaminase activity. *Curr. Biol.* **15**, 166–170 (2005).

9. F. Navarro, B. Bollman, H. Chen, R. König, Q. Yu, K. Chiles, N. R. Landau, Complementary function of the two catalytic domains of APOBEC3G. *Virology* **333**, 374–386 (2005).

10. K.-M. Chen, E. Harjes, P. J. Gross, A. Fahmy, Y. Lu, K. Shindo, R. S. Harris, H. Matsuo, Structure of the DNA deaminase domain of the HIV-1 restriction factor APOBEC3G. *Nature* **452**, 116–119 (2008).

11. L. G. Holden, C. Prochnow, Y. P. Chang, R. Bransteitter, L. Chelico, U. Sen, R. C. Stevens, M. F. Goodman, X. S. Chen, Crystal structure of the anti-viral APOBEC3G catalytic domain and functional implications. *Nature* **456**, 121–124 (2008).

12. Y. Iwatani, D. S. B. Chan, F. Wang, K. Stewart-Maynard, W. Sugiura, A. M. Gronenborn, I. Rouzina, M. C. Williams, K. Musier-Forsyth, J. G. Levin, Deaminase-independent inhibition of HIV-1 reverse transcription by APOBEC3G. *Nucleic Acids Res.* **35**, 7096–7108 (2007).

13. D. Pollpeter, M. Parsons, A. E. Sobala, S. Coxhead, R. D. Lang, A. M. Bruns, S. Papaioannou, J. M. McDonnell, L. Apolonia, J. A. Chowdhury, C. M. Horvath, M. H. Malim, Deep sequencing of HIV-1 reverse transcripts reveals the multifaceted antiviral functions of APOBEC3G. *Nat. Microbiol.* **3**, 220–233 (2018).

14. V. B. Soros, W. Yonemoto, W. C. Greene, Newly synthesized APOBEC3G is incorporated into HIV virions, inhibited by HIV RNA, and subsequently activated by RNase H. *PLOS Pathog.* **3**, e15 (2007).

15. H. Huthoff, F. Autore, S. Gallois-Montbrun, F. Fraternali, M. H. Malim, RNA-dependent oligomerization of APOBEC3G is required for restriction of HIV-1. *PLOS Pathog.* **5**, e1000330 (2009).

16. K. Bélanger, M. Savoie, M. C. Rosales Gerpe, J.-F. Couture, M.-A. Langlois, Binding of RNA by APOBEC3G controls deamination-independent restriction of retroviruses. *Nucleic Acids Res.* **41**, 7438–7452 (2013).

17. A. M. Sheehy, N. C. Gaddis, J. D. Choi, M. H. Malim, Isolation of a human gene that inhibits HIV-1 infection and is suppressed by the viral Vif protein. *Nature* **418**, 646–650 (2002).

18. M. Marin, K. M. Rose, S. L. Kozak, D. Kabat, HIV-1 Vif protein binds the editing enzyme APOBEC3G and induces its degradation. *Nat. Med.* **9**, 1398–1403 (2003).

19. A. M. Sheehy, N. C. Gaddis, M. H. Malim, The antiretroviral enzyme APOBEC3G is degraded by the proteasome in response to HIV-1 Vif. *Nat. Med.* **9**, 1404–1407 (2003).

20. X. Yu, Y. Yu, B. Liu, K. Luo, W. Kong, P. Mao, X.-F. Yu, Induction of APOBEC3G ubiquitination and degradation by an HIV-1 Vif-Cul5-SCF complex. *Science* **302**, 1056–1060 (2003).

21. S. Jäger, D. Y. Kim, J. F. Hultquist, K. Shindo, R. S. La Rue, E. Kwon, M. Li, B. D. Anderson, L. Yen, D. Stanley, C. Mahon, J. Kane, K. Franks-Skiba, P. Cimermancic, A. Burlingame, A. Sali, C. S. Craik, R. S. Harris, J. D. Gross, N. J. Krogan, Vif hijacks CBF-β to degrade APOBEC3G and promote HIV-1 infection. *Nature* **481**, 371–375 (2011).

22. W. Zhang, J. Du, S. L. Evans, Y. Yu, X.-F. Yu, T-cell differentiation factor CBF-β regulates HIV-1 Vif-mediated evasion of host restriction. *Nature* **481**, 376–379 (2011).

23. H. Yang, F. Ito, A. D. Wolfe, S. Li, N. Mohammadzadeh, R. P. Love, M. Yan, B. Zirkle, A. Gaba, L. Chelico, X. S. Chen, Understanding the structural basis of HIV-1 restriction by the full length double-domain APOBEC3G. *Nat. Commun.* **11**, 632 (2020).

24. A. Maiti, W. Myint, K. A. Delviks-Frankenberry, S. Hou, T. Kanai, V. Balachandran, C. Sierra Rodriguez, R. Tripathi, N. Kurt Yilmaz, V. K. Pathak, C. A. Schiffer, H. Matsuo, Crystal structure of a soluble APOBEC3G variant suggests ssDNA to bind in a channel that extends between the two domains. *J. Mol. Biol.* **432**, 6042–6060 (2020).

25. Y. Guo, L. Dong, X. Qiu, Y. Wang, B. Zhang, H. Liu, Y. Yu, Y. Zang, M. Yang, Z. Huang, Structural basis for hijacking CBF-β and CUL5 E3 ligase complex by HIV-1 Vif. *Nature* **505**, 229–233 (2014).

26. F. C. Azimi, J. E. Lee, Structural perspectives on HIV-1 Vif and APOBEC3 restriction factor interactions. *Protein Sci.* **29**, 391–406 (2020).

27. X. Xiao, S.-X. Li, H. Yang, X. S. Chen, Crystal structures of APOBEC3G N-domain alone and its complex with DNA. *Nat. Commun.* **7**, 12193 (2016).

28. R. Mariani, D. Chen, B. Schröfelbauer, F. Navarro, R. König, B. Bollman, C. Münk, H. Nymark-McMahon, N. R. Landau, Species-specific exclusion of APOBEC3G from HIV-1 virions by Vif. *Cell* **114**, 21–31 (2003).

29. M. Letko, T. Booiman, N. Kootstra, V. Simon, M. Ooms, Identification of the HIV-1 Vif and human APOBEC3G protein interface. *Cell Rep.* **13**, 1789–1799 (2015).

30. H. P. Bogerd, B. P. Doehle, H. L. Wiegand, B. R. Cullen, A single amino acid difference in the host APOBEC3G protein controls the primate species specificity of HIV type 1 virion infectivity factor. *Proc. Natl. Acad. Sci. U.S.A.* **101**, 3770–3774 (2004).

31. B. Schröfelbauer, D. Chen, N. R. Landau, A single amino acid of APOBEC3G controls its species-specific interaction with virion infectivity factor (Vif). *Proc. Natl. Acad. Sci. U.S.A.* **101**, 3927–3932 (2004).

32. H. Xu, E. S. Svarovskaia, R. Barr, Y. Zhang, M. A. Khan, K. Strebel, V. K. Pathak, A single amino acid substitution in human APOBEC3G antiretroviral enzyme confers resistance to HIV-1 virion infectivity factor-induced depletion. *Proc. Natl. Acad. Sci. U.S.A.* **101**, 5652–5657 (2004).

33. B. Mangeat, P. Turelli, S. Liao, D. Trono, A single amino acid determinant governs the species-specific sensitivity of APOBEC3G to Vif action. *J. Biol. Chem.* **279**, 14481–14483 (2004).

34. T. Kouno, E. M. Luengas, M. Shigematsu, S. M. D. Shandilya, J.Y. Zhang, L. Chen, M. Hara, C. A. Schiffer, R. S. Harris, H. Matsuo, Structure of the Vif-binding domain of the antiviral enzyme APOBEC3G. *Nat. Struct. Mol. Biol.* **22**, 485–491 (2015).

35. D. C. Scott, D. Y. Rhee, D. M. Duda, I. R. Kelsall, J. L. Olszewski, J. A. Paulo, A. de Jong, H. Ovaa, A. F. Alpi, J. W. Harper, B. A. Schulman, Two distinct types of E3 ligases work in unison to regulate substrate ubiquitylation. *Cell* **166**, 1198–1214.e24 (2016).

36. R. Hüttenhain, J. Xu, L. A. Burton, D. E. Gordon, J. F. Hultquist, J. R. Johnson, L. Satkamp, J. Hiatt, D. Y. Rhee, K. Baek, D. C. Crosby, A. D. Frankel, A. Marson, J Wade Harper, A. F. Alpi, B. A. Schulman, J. D. Gross, N. J. Krogan, ARIH2 Is a Vif-dependent regulator of CUL5-mediated APOBEC3G degradation in HIV infection. *Cell Host Microbe* **26**, 86–99.e7 (2019).

37. S. Kostrhon, J. R. Prabu, K. Baek, D. Horn-Ghetko, S. von Gronau, M. Klügel, J. Basquin, A. F. Alpi, B. A. Schulman, CUL5-ARIH2 E3-E3 ubiquitin ligase structure reveals cullin-specific NEDD8 activation. *Nat. Chem. Biol.* **17**, 1075–1083 (2021).

38. Y. Iwatani, D. S. B. Chan, L. Liu, H. Yoshii, J. Shibata, N. Yamamoto, J. G. Levin, A. M. Gronenborn, W. Sugiura, HIV-1 Vif-mediated ubiquitination/degradation of APOBEC3G involves four critical lysine residues in its C-terminal domain. *Proc. Natl. Acad. Sci. U.S.A.* **106**, 19539–19544 (2009).

39. J. A. Bohn, K. Thummar, A. York, A. Raymond, W. C. Brown, P. D. Bieniasz, T. Hatziioannou, J. L. Smith, APOBEC3H structure reveals an unusual mechanism of interaction with duplex RNA. *Nat. Commun.* **8**, 1021 (2017).

40. N. M. Shaban, K. Shi, K. V. Lauer, M. A. Carpenter, C. M. Richards, D. Salamango, J. Wang, M. W. Lopresti, S. Banerjee, R. Levin-Klein, W. L. Brown, H. Aihara, R. S. Harris, The antiviral and cancer genomic DNA deaminase APOBEC3H is regulated by an RNA-mediated dimerization mechanism. *Mol. Cell* **69**, 75–86.e9 (2018).

41. T. Matsuoka, T. Nagae, H. Ode, H. Awazu, T. Kurosawa, A. Hamano, K. Matsuoka, A. Hachiya, M. Imahashi, Y. Yokomaku, N. Watanabe, Y. Iwatani, Structural basis of chimpanzee APOBEC3H dimerization stabilized by double-stranded RNA. *Nucleic Acids Res.* **46**, 10368–10379 (2018).

42. R. M. Kaake, I. Echeverria, S. J. Kim, J. von Dollen, N. M. Chesarino, Y. Feng, C. Yu, H. Ta, L. Chelico, L. Huang, J. Gross, A. Sali, N. J. Krogan, Characterization of an A3G-Vif_{HIV-1}-CRL5-CBFβ structure using a cross-linking mass spectrometry pipeline for integrative modeling of host-pathogen complexes. *Mol. Cell. Proteomics* **20**, 100132 (2021).

43. Y. Hu, B. A. Desimmie, H. C. Nguyen, S. J. Ziegler, T. C. Cheng, J. Chen, J. Wang, H. Wang, K. Zhang, V. K. Pathak, Y. Xiong, Structural basis of antagonism of human APOBEC3F by HIV-1 Vif. *Nat. Struct. Mol. Biol.* **26**, 1176–1183 (2019).

44. R. A. Russell, V. K. Pathak, Identification of two distinct human immunodeficiency virus type 1 Vif determinants critical for interactions with human APOBEC3G and APOBEC3F. *J. Virol.* **81**, 8201–8210 (2007).

45. G. Chen, Z. He, T. Wang, R. Xu, X.-F. Yu, A patch of positively charged amino acids surrounding the human immunodeficiency virus type 1 Vif SLVx4Yx9Y motif influences its interaction with APOBEC3G. *J. Virol.* **83**, 8674–8682 (2009).

46. L. Apolonia, R. Schulz, T. Curk, P. Rocha, C. M. Swanson, T. Schaller, J. Ule, M. H. Malim, Promiscuous RNA binding ensures effective encapsidation of APOBEC3 proteins by HIV-1. *PLOS Pathog.* **11**, e1004609 (2015).

47. A. York, S. B. Kutluay, M. Errando, P. D. Bieniasz, The RNA binding specificity of human APOBEC3 proteins resembles that of HIV-1 nucleocapsid. *PLOS Pathog.* **12**, e1005833 (2016).

48. W. Selleck, S. Tan, Recombinant protein complex expression in *E. coli*. *Curr. Protoc. Protein Sci.* **Chapter 5**, Unit 5 21 (2008).

49. A. Punjani, J. L. Rubinstein, D. J. Fleet, M. A. Brubaker, cryoSPARC: Algorithms for rapid unsupervised cryo-EM structure determination. *Nat. Methods* **14**, 290–296 (2017).

50. J. Tholen, M. Razew, F. Weis, W. P. Galej, Structural basis of branch site recognition by the human spliceosome. *Science* **375**, 50–57 (2022).

51. A. Punjani, H. Zhang, D. J. Fleet, Non-uniform refinement: Adaptive regularization improves single-particle cryo-EM reconstruction. *Nat. Methods* **17**, 1214–1221 (2020).

52. S. Chen,G. McMullan, A. R. Faruqi, G. N. Murshudov, J. M. Short, S. H.W. Scheres, R. Henderson, High-resolution noise substitution to measure overfitting and validate resolution in 3D structure determination by single particle electron cryomicroscopy. *Ultramicroscopy* **135**, 24–35 (2013).

53. E. F. Pettersen,T. D. Goddard, C. C. Huang, G. S. Couch, D. M. Greenblatt, E. C. Meng, T. E. Ferrin, UCSF Chimera—A visualization system for exploratory research and analysis. *J. Comput. Chem.* **25**, 1605–1612 (2004).

54. P. D. Adams,P. V. Afonine, G. Bunkóczi, V. B. Chen, I. W. Davis, N. Echols, J. J. Headd, L.-W. Hung, G. J. Kapral, R. W. Grosse-Kunstleve, A. J. McCoy, N. W. Moriarty, R. Oeffner, R. J. Read, D. C. Richardson, J. S. Richardson, T. C. Terwilliger, P. H. Zwart, PHENIX: A comprehensive Python-based system for macromolecular structure solution. *Acta Crystallogr. D Biol. Crystallogr.* **66**, 213–221 (2010).

55. P. V. Afonine,R. W. Grosse-Kunstleve, N. Echols, J. J. Headd, N. W. Moriarty, M. Mustyakimov, T. C. Terwilliger, A. Urzhumtsev, P. H. Zwart, P. D. Adams, Towards automated crystallographic structure refinement with phenix.refine. *Acta Crystallogr. D Biol. Crystallogr.* **68**, 352–367 (2012).

56. P. Emsley, B. Lohkamp, W. G. Scott, K. CoWTan, Features and development of Coot. *Acta Crystallogr. D Biol. Crystallogr.* **66**, 486–501 (2010).

57. P. V. Afonine,B. P. Klaholz, N. W. Moriarty, B. K. Poon, O. V. Sobolev, T. C. Terwilliger, P. D. Adams, A. Urzhumtsev, New tools for the analysis and validation of cryo-EM maps and atomic models. *Acta Crystallogr. D Struct. Biol.* **74**, 814–840 (2018).

58. T. D. Goddard,C. C. Huang, E. C. Meng, E. F. Pettersen, G. S. Couch, J. H. Morris, T. E. Ferrin, UCSF ChimeraX: Meeting modern challenges in visualization and analysis. *Protein Sci.* **27**, 14–25 (2018).

59. K. M. Williams,S. Qie, J. H. Atkison, S. Salazar-Arango, J. Alan Diehl, S. K. Olsen, Structural insights into E1 recognition and the ubiquitin-conjugating activity of the E2 enzyme Cdc34. *Nat. Commun.* **10**, 3296 (2019).

60. K. Baek,D. T. Krist, J. R. Prabu, S. Hill, M. Klügel, L.-M. Neumaier, S. von Gronau, G. Kleiger, B. A. Schulman, NEDD8 nucleates a multivalent cullin-RING-UBE2D ubiquitin ligation assembly. *Nature* **578**, 461–466 (2020).
